# Limitation of Maximal Heart Rate in Hypoxia: Mechanisms and Clinical Importance

**DOI:** 10.3389/fphys.2018.00972

**Published:** 2018-07-23

**Authors:** Laurent Mourot

**Affiliations:** ^1^EA 3920 Prognostic Markers and Regulatory Factors of Cardiovascular Diseases and Exercise Performance, Health, Innovation Platform, University of Franche-Comté, Besançon, France; ^2^Tomsk Polytechnic University, Tomsk, Russia

**Keywords:** exercise, training, altitude, disease, cardiovascular, physiology, rehabilitation

## Abstract

The use of exercise intervention in hypoxia has grown in popularity amongst patients, with encouraging results compared to similar intervention in normoxia. The prescription of exercise for patients largely rely on heart rate recordings (percentage of maximal heart rate (HR_max_) or heart rate reserve). It is known that HR_max_ decreases with high altitude and the duration of the stay (acclimatization). At an altitude typically chosen for training (2,000-3,500 m) conflicting results have been found. Whether or not this decrease exists or not is of importance since the results of previous studies assessing hypoxic training based on HR may be biased due to improper intensity. By pooling the results of 86 studies, this literature review emphasizes that HR_max_ decreases progressively with increasing hypoxia. The dose–response is roughly linear and starts at a low altitude, but with large inter-study variabilities. Sex or age does not seem to be a major contributor in the HR_max_ decline with altitude. Rather, it seems that the greater the reduction in arterial oxygen saturation, the greater the reduction in HRmax, due to an over activity of the parasympathetic nervous system. Only a few studies reported HR_max_ at sea/low level and altitude with patients. Altogether, due to very different experimental design, it is difficult to draw firm conclusions in these different clinical categories of people. Hence, forthcoming studies in specific groups of patients are required to properly evaluate (1) the HR_max_ change during acute hypoxia and the contributing factors, and (2) the physiological and clinical effects of exercise training in hypoxia with adequate prescription of exercise training intensity if based on heart rate.

## Introduction

Hypoxia is defined as a reduction in the amount of oxygen (O_2_) available to any cell, tissue, or organism (Semenza, 2009). Environmental hypoxia (natural as at terrestrial altitude or artificial using chamber or other devices producing normobaric or hypobaric hypoxia) reduces arterial oxygen saturation and induces hypoxia in peripheral skeletal muscle (Hoppeler et al., 2008; Vogt and Hoppeler, 2010; Girard et al., 2017). This reduction impairs endurance performance and maximal oxygen consumption (VO_2max_) both in hypobaric and normobaric environments (Fagraeus et al., 1973; Knuttgen and Saltin, 1973; Cerretelli, 1976; Shephard et al., 1988; Cymerman et al., 1989; Koistinen et al., 1995; Peltonen et al., 1995; Ferretti et al., 1997; Calbet et al., 2003a; Angermann et al., 2006; Mollard et al., 2007c) even if the difference between these two environments is debated (Millet et al., 2012; Mounier and Brugniaux, 2012; Beidleman et al., 2014). Research teams have examined the effects of this reduction on endurance performance (Beidleman et al., 2014; Hamlin et al., 2018), and the benefits deriving from exposure to hypoxia on sea level or altitude performance (Bonetti and Hopkins, 2009; Millet et al., 2010; Vogt and Hoppeler, 2010).

**Figure 1:**
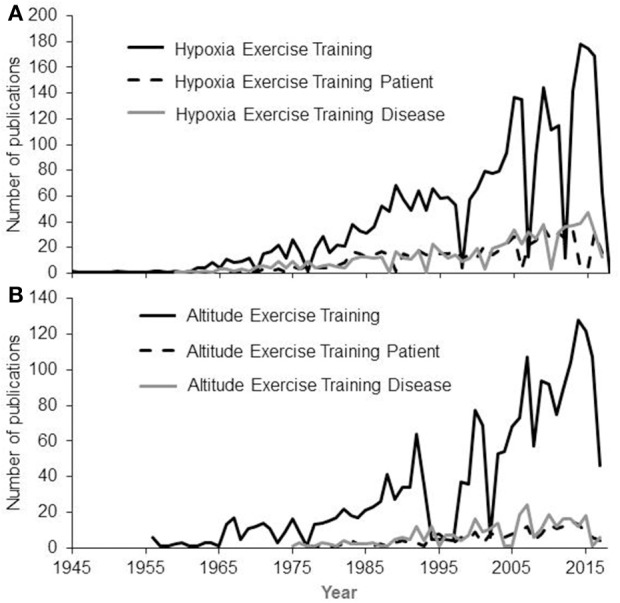
Numbers of publications having the Key Words Hypoxia **(A)** OR Altitude **(B)** AND Exercise Training AND Patient OR Disease. A continuous increase is observed since the first works in 1945 based on searches made on PubMed in March 2018.

On the other hand, epidemiologic studies have shown that living at altitude (1,500-3,500 m elevation) is associated with lower prevalence of diabetes, obesity and hypertension than living at lower altitude, even after adjusting for multiple risk factors and potential confounders (Sharma, 1990; Woolcott et al., 2014, 2016; Díaz-Gutiérrez et al., 2016). Intermittent exposure to hypoxia at rest is thus considered as an alternative non-pharmacological therapy (Serebrovskaya and Xi, 2016; Leone and Lalande, 2017), even though a thorough evaluation of its effectiveness has yet to be performed (Wilson et al., 2018). Based on these observations, several authors have suggested that exercise intervention in a hypoxic environment should be used with patients (Chapman et al., 1999; Millet et al., 2016b; Hobbins et al., 2017). Accordingly, the number of studies dedicated to the evaluation of exercise training in hypoxia has dramatically increased in the past few years (Figure 1).

Even if the discussion is still open and an important role seems to be played by the chosen intensity (Noordhof et al., 2013), submaximal oxygen uptake (VO_2_) at a specific power output is similar at sea level and altitude (Clark et al., 2007). As VO_2max_ progressively declines with an increase in elevation (Wehrlin and Hallen, 2006), the relative difficulty of exercising at a specific absolute power output will progressively increase as elevation increases. To induce specific adaptations through exercise training in hypoxia, various exercise training programs can be used (Girard et al., 2017) where, in some cases, hypoxic-induced adaptive responses would result from reduced absolute training intensity (Hobbins et al., 2017). Hence, exercise training in hypoxic, or “living low-training high,” appears to be a promising method of optimizing training such that participants (e.g., patients with cardiovascular disease, obese people or elderly) receive the maximal metabolic and cardiovascular benefit whilst minimizing injury risk (Bailey et al., 2000; Haufe et al., 2008).

Most studies evaluating the effects of exercise training in hypoxia have used a control group training in normoxia. In healthy subjects, the total work performed (Faiss et al., 2015), or percentage of peak power output achieved in the corresponding environment (Wang et al., 2010) have been used to match the exercise intensity between the experimental and control groups. However, in studies involving patients, the intensity of the training program is often based on the percentage of maximal heart rate (HR_max_; Netzer et al., 2008; Kong et al., 2014; Park and Lim, 2017) or heart rate reserve (HRR; Greie et al., 2006; Mao et al., 2011; Schreuder et al., 2014; Gutwenger et al., 2015). Heart rate (HR) monitoring is practically useful and very common with athletes and patients (Achten and Jeukendrup, 2003; Casillas et al., 2017). But, as for the debate on the correct metric to evaluate the hypoxic dose (Garvican-Lewis et al., 2016; Millet et al., 2016a), no consensus yet exists on the best way to tailor exercise training intensity in hypoxia. In a recent review dealing with exercise training in hypoxia for overweight/obese subjects, the authors suggested setting the intensity at 60-70% of HR_max_ (Hobbins et al., 2017). With healthy people, an intensity target between 75 and 95% of HR_max_ was proposed (Vogt and Hoppeler, 2010).

However, the authors did not mention if HR_max_ used in the calculation of the target intensity is the HR_max_ obtained at sea level or at the considered altitude. This is of importance as HR_max_ may be altered in hypoxia. It is well established that HR_max_ is decreased during prolonged (several days or weeks) exposure to a high altitude such as encountered during mountaineering (Christensen and Forbes, 1937; Richalet et al., 1992; Lundby et al., 2001b). On the contrary, the change under acute hypoxic exposure is still debated, particularly at moderate levels of hypoxia. Studies have shown that HR_max_ is (e.g., Grataloup et al., 2007) or is not (e.g., Gallagher et al., 2015) significantly reduced in acute hypobaric and normobaric hypoxia (see Supplementary Table S1). Some researchers suggest that a decreased HR_max_ with altitude is specific, with a threshold around 2,000-3,500 m (Dill et al., 1966; Martin and O'Kroy, 1993; Benoit et al., 2003; Calbet et al., 2003a; Fukuda et al., 2010; Gallagher et al., 2015), i.e., typically the altitude chosen for training/rehabilitation purpose (see Supplementary Table S2). However, the exact altitude at which HR_max_ is decreased is not well known and it is likely that, similar to VO_2max_, the decrement in HR_max_ starts at low altitude (below 1,000 m; Gore et al., 1996, 1997; Robergs et al., 1997). Several factors may account for this lack of a consistently observed HR_max_ decline within increasing altitude such as exercise-induced hypoxemia (Benoit et al., 2003; Grataloup et al., 2007), training status (Kjaer et al., 1988; Benoit et al., 2003; Dufour et al., 2006; Roels et al., 2007; Mollard et al., 2007b) or sex (Shephard et al., 1988; Robergs et al., 1997) as well as the robustness of statistical analyses (usually small numbers of participants and high inter-individual variability in the HR response). Also, most of the previous data were obtained with healthy young participants, and thus extrapolation of this to older people or patients with limited capacities is unknown.

As shown in Figure 1, the number of publications related to exercise training in hypoxia amongst both healthy subjects and patients is increasing, highlighting the scientific and clinical interest of such exercise training. Hence, this literature review presents an examination of the available literature on HR_max_ decrement with increasing hypoxia in both healthy subjects and patients, with the main aim of providing a general understanding of the use of HR_max_ as a benchmark for exercise training prescription in hypoxia.

## Method

A literature search was made up until March 2018 using the following databases: Pubmed, ScienceDirect, Scopus, SportsDiscus and Web of Science. Search terms were used to restrict studies to those involving humans under hypoxic conditions, including a combination of either “altitude” or “hypoxia” or “oxygen fraction” or “oxygen delivery” and each of the following: “exercise,” “exercise training,” “Live Low-Train High,” “intermittent hypoxia,” “incremental,” “GXT,” “graded,” “heart rate,” “exhaustion,” “patient,” “disease.”

Each title, abstract and full text were assessed for relevance to the topic and selected if they met the following inclusion criteria: involving humans; normoxia and hypobaric or normobaric hypoxia conditions; hypoxic level expressed as altitude (m) and/or inspired oxygen fraction (%). A classification was first completed to select articles reporting HR_max_ in both hypoxia and normoxia. To do so, the common criteria to assess that maximal intensity was reached at the end of the incremental test to exhaustion were considered (Howley et al., 1995) except for older ones within which the authors′ statement of “maximal intensity” was deemed adequate. Also, only acute exposure to hypoxia, i.e., the maximal test should have been performed within 8h of exposure to the hypoxic environment was taken into consideration. A second classification was made including studies using HR to monitor the training intensity. References of articles that fulfilled the criteria were also scanned for further relevant studies that were included if they met the inclusion criteria.

The initial search yielded a total of 2,077 publications. From these, 1,991 publications were excluded. Finally HR_max_ data were collated from 81 publications involving healthy subjects and 5 publications involving patients (Supplementary Table S1). In five of the reviewed articles, the data were not reported in the text/Tables but in figures. In these cases, the data were extracted using the Digitizelt software (v2.2, BormiSoft2, Braunschweig, Germany). Thirteen publications with healthy subjects and 9 with patients compared exercise training in hypoxia vs. exercise training in normoxia matching the intensity with HR (Supplementary Table S2).

Due to the lack of standardization of study designs (the most important of which being the hypoxic stimulus, but also the type of disease, the age or gender of participants, the type of incremental test), a systematic assessment of hypoxic intervention was not done.

## Decrease in HR_max_ in acute hypoxia: a universal response?

**Figure 2:**
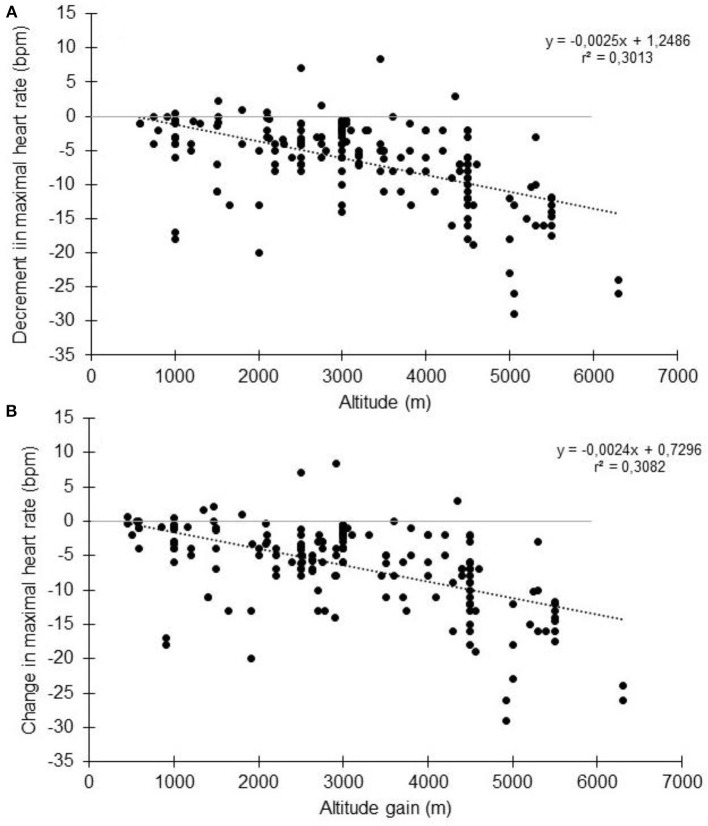
Change in maximal heart rate with altitude **(A)** or altitude gain **(B)**. Each data point represents the average value from the relevant group, as presented in previously published studies (references in Supplementary Table S1).

**Figure 3:**
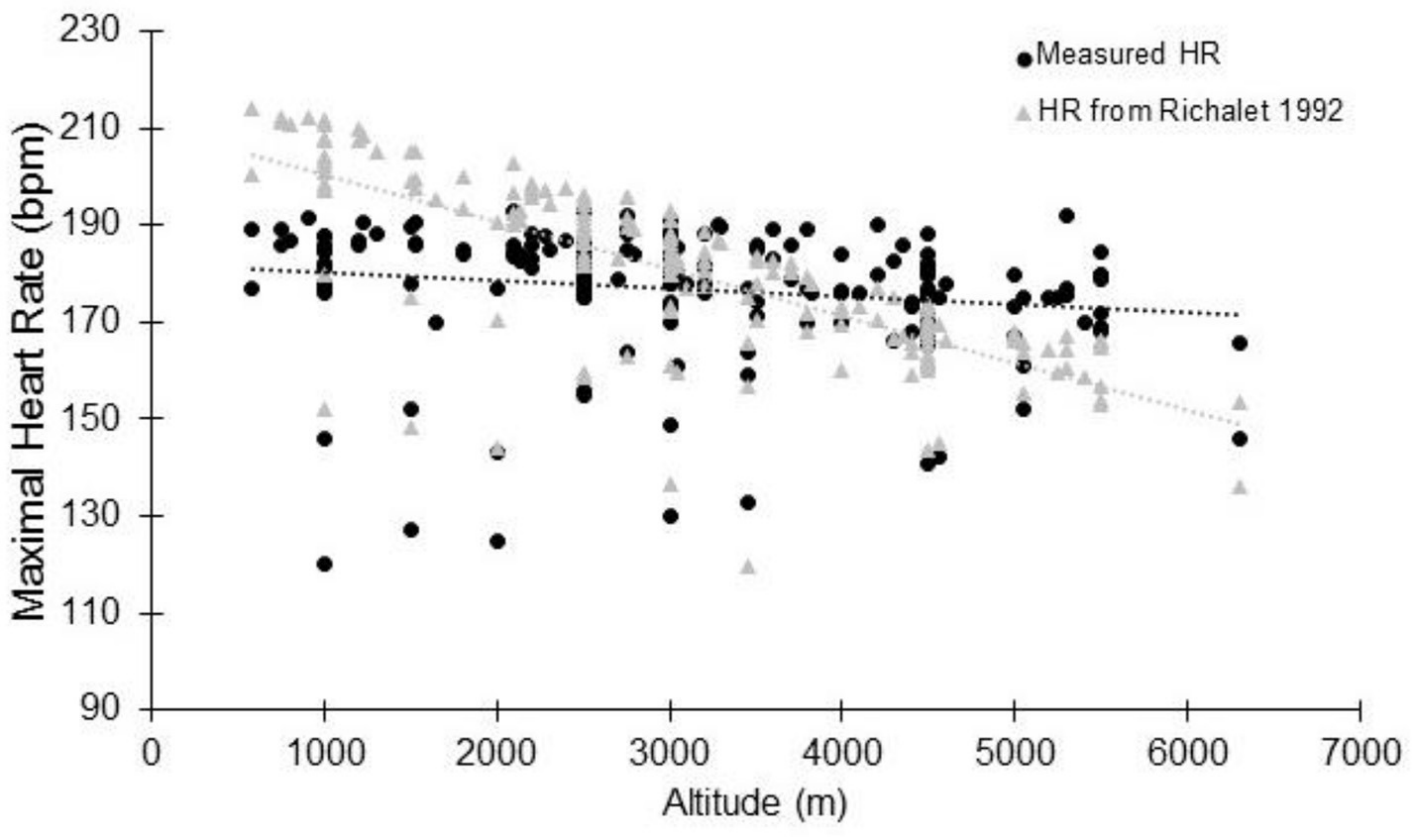
Maximal heart rate at altitude: comparison between the measured data (see Supplementary Table S1) and data modelised according to Richalet (1990). Each data point represents the average value from the relevant group, as presented in previously published studies (references in Supplementary Table S1).

In high altitude (as defined elsewhere Bärtsch and Saltin, 2008), previous studies have shown that the decrement in VO_2max_ could not be fully explained by the reduction in arterial oxygen content (CaO_2_) (Calbet et al., 2003a). It has been calculated that at 5,300 m, the VO_2max_ decrease could be explained by a two-thirds reduction in CaO_2_ and one-third reduction in peak muscle blood flow and cardiac output (Calbet et al., 2003a). When subjects are exposed to acute hypoxia, the lower arterial oxygen saturation (*S*aO_2_) is usually associated with lower HR_max_ (Benoit et al., 2003). Reduction in HR_max_ with increasing altitude and with time spent in altitude has been observed for a long time (Christensen and Forbes, 1937), with a HR_max_ of 127 bpm reported at an altitude equivalent to 8,848 m (Cymerman et al., 1989). During acute exposure, HR_max_ has long been believed to remain stable below simulated altitudes of 4,500 m (Cerretelli et al., 1967; Saltin et al., 1968; Fagraeus et al., 1973; Knuttgen and Saltin, 1973; Young et al., 1982; Escourrou et al., 1984; Bouissou et al., 1986; Kjaer et al., 1988; Lawler et al., 1988; Shephard et al., 1988; Hughson et al., 1995). In contrast, other studies have strongly argued in favor of decreased HR_max_ during acute hypoxia (Dill et al., 1966; Drinkwater et al., 1979; Roach et al., 1996; Lundby and van Hall, 2001) even at lower altitudes. Lundby and van Hall suggested that HR_max_ remains stable up to 3,100-3,300 m with a linear decrease thereafter, but this result was obtained in only 5 healthy subjects without a thorough evaluation below that threshold (Lundby and van Hall, 2001). The current literature research involving humans performing maximal incremental tests to exhaustion showed that HR_max_ reduces with acute normobaric or hypobaric hypoxia (Supplementary Table S1 and Figure 2, showing group average data). When the data are pooled together, the overall tendency is that HR_max_ starts to decrease as soon as altitude increase. This is accordance with the observation made at as low as 600-700 m above sea level (Gore et al., 1996). No clear threshold in the decrement is observed. Instead, a roughly linear trend seems to exist, but there is large inter-study variation. Together with the large inter-individual variation within studies, this likely explains why the reported differences in the literature are not always statistically significant (Supplementary Table S1). It should be acknowledged that likely more than one mathematical model could fit the data, as observed in other circumstances (Garvican-Lewis et al., 2016). Instead of absolute altitude *per se*, the altitude gain between the two incremental tests is likely to be more meaningful (Figure 2B). In that case, the relationship between the two variables could be modeled by the following equation: HR_max_ (bpm) = −0.0024 × altitude (m) + 0.73. Hence, a 1,000 m gain will reduce HR_max_ by 1.7 bpm. This is in agreement with, e.g., the 1.9 bpm per 1,000 m found in young subjects by Wehrlin et al. and with the 9 bpm decrease of HR_max_ observed at 4,300 m (Lundby et al., 2001a). It implies that if participants usually train at 200 m and performed a hypoxic training session at 2,500 m, the expected decrement in HR_max_ will be 5 bpm. Richalet (1990) proposed an equation that describes the decrease in the chronotropic drive during hypoxia to predict HR_max_: y (% of sea-level HR_max_) = 116–0.0057x (x = altitude in meters). Compared to our findings, this model tends to underestimate the decrease at low altitudes and overestimate the decrease at high altitudes (Figure 3). But the original data used to obtain the model are somewhat different, and includes chronic hypoxic exposure, which is known to decrease HR_max_ (Favret and Richalet, 2007).

### Effect of age

**Figure 4:**
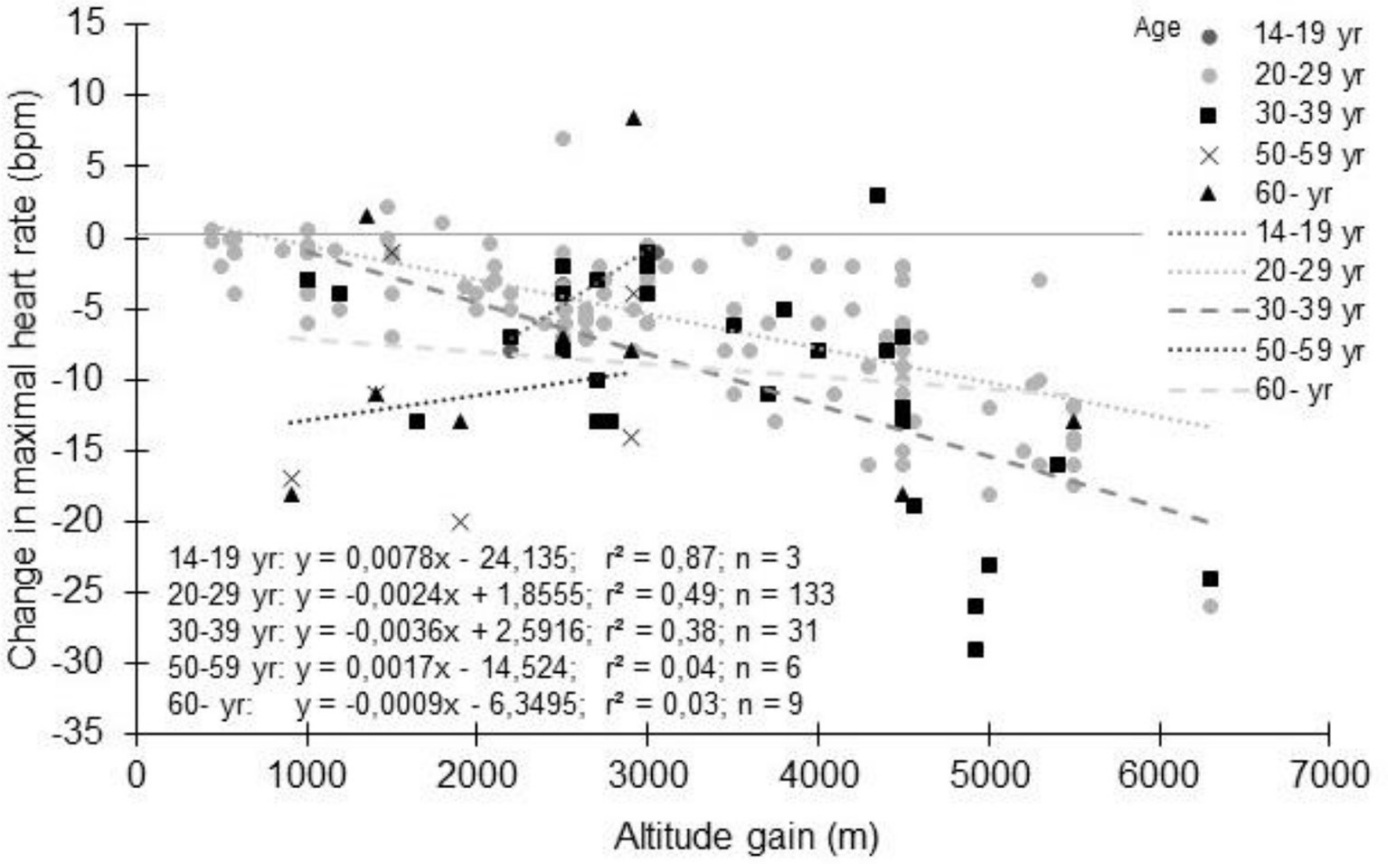
Change in maximal heart rate with altitude: Effect of age. Each data point represents the average value from the relevant group, as presented in previously published studies (references in Supplementary Table S1).

The large variability in the decrement in HR_max_ with altitude could be explained by several factors. Most of the studies involved healthy young adults (between 20 and 39 years old as shown in Figure 4). Obviously, the general trends show a progressive decrease in HR_max_ with altitude whatever the age groups, without marked differences (Puthon et al., 2017). A trend toward a slightly greater decrease is observed in 30-39 years vs. 20-29 years. In Figure 4, 14-19 years and 50-59 years age groups show different behaviors than 20–29 years and 30–39 years age groups, but likely due to small sample sizes and to the fact that for these ages groups, participants were patients for whom only few data are available (Supplementary Table S1). Hence, if HR_max_ decreases with age (Lhuissier et al., 2012), age *per se* does not seem to alter the decrement in HR_max_ with acute hypoxia (Puthon et al., 2017), but this needs to be confirmed by specifically designed studies. It also suggests that the hypoxia effects will be relatively greater for an older subject compared to a younger one. For example at 2,500m, using the equation presented in Figure 2, the decrement in HR_max_ is 5 bpm (absolute value). For a 20 years old subject, this represents a decrease of 2.5% (assuming HR_max_ = 200 bpm in normoxia). For a 70 years old subject, this represents 3.3% (assuming HR_max_ = 150 bpm in normoxia). As older subjects already have cardiac limitations, this further decrease may result in an insufficient HR to maintain performance and a tendency to reach exercise intolerance at the suggested exercise target HR.

### Effect of sex

Most of the time, both male and female are mixed in the studies and only a few studies specifically focused on possible sex differences. Both females and males experience a decrease in HR_max_ with altitude (Horvath et al., 1988; Shephard et al., 1988; Gore et al., 1997; Mollard et al., 2007b) without marked difference between sexes.

### Effect of fitness/training status

**Figure 5:**
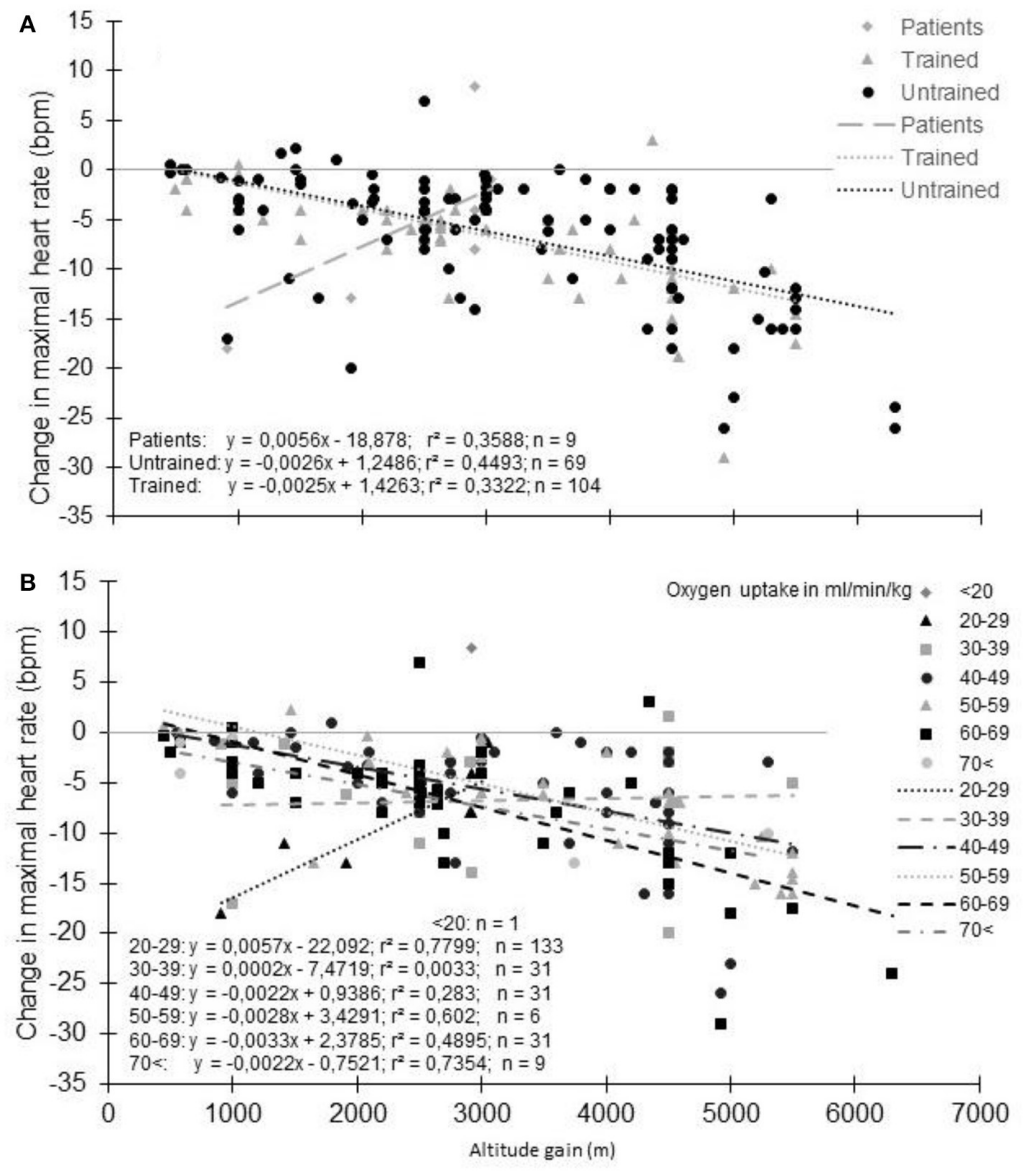
Decrement of maximal heart rate with altitude: Effect of physical fitness (**A**) groups based on subjects' training characteristics. (**B**) groups based on maximal oxygen uptake). Each data point represents the average value from the relevant group, as presented in previously published studies (references in Supplementary Table S1).

The magnitude of the HR_max_ decrease is more likely dependent on the training status. When subjects were grouped based on their training characteristics (i.e., healthy untrained vs. trained subjects as characterized by the authors of the studies), the decrement in HR_max_ appears a little more pronounced for untrained than trained subjects (Figure 5A); HR_max_ (bpm) = −0.0026 × altitude (m) + 1.2486 and HR_max_ (bpm) = −0.0025 × altitude (m) + 1.4263, respectively. However, this observation is based on studies using different testing and hypoxic conditions and should be viewed with caution. Also, the definition of the “untrained” and “trained” states by the authors has not been harmonized as it should be (De Pauw et al., 2013), so that the same VO_2max_ was considered “trained” in some studies and “untrained” in others when VO_2max_ was reported. When the decrease in HR_max_ with altitude takes VO_2max_ into account (Figure 5B), the higher the VO_2max_, the greater the decrease in HR_max_. This is consistent with the results in the few studies that directly compared trained and untrained subjects. They usually report a larger decrease in HR_max_ (Kjaer et al., 1988; Martin and O'Kroy, 1993; Benoit et al., 2003; Marconi et al., 2004; Mollard et al., 2007b,c) even if not universally observed (Lawler et al., 1988; Gore et al., 1996; Ferretti et al., 1997; Ofner et al., 2014). Hence, HR_max_ reduction with acute hypoxia appears to depend on the training status, even if the reason for this decrement remains unclear (Mollard et al., 2007b). It should be noted that athletes involved in studies reporting results from incremental tests to exhaustion in hypoxia are usually aerobically-trained athletes. It is currently unknown whether another type of training background could alter the HR_max_ response to hypoxia, while exercise training in hypoxia is more and more commonly used to improve intermittent and strength performance (Millet et al., 2010; Saeed et al., 2012; Brocherie et al., 2017; Feriche et al., 2017; Girard et al., 2017), even if for these activities HR_max_ is less meaningful.

### Effect of pathology

In patients/individual with a low VO_2max_ (14-39 ml/min/kg), the trend is completely different with a paradoxical grseater decrease in HR_max_ at the lowest reported altitude (Supplementary Table S1; Figure 5). However, HR_max_ in normoxia and hypoxia were reported in only 6 studies (9 different groups/altitude) limiting the relevance of this finding. Interestingly, in one study with heart failure patients, an unexpected increase in HR_max_ of 8 bpm was reported at 3454 m compared to 540 m (Schmid et al., 2015). The patients may likely have “learned” the incremental test procedure, as all tests in normoxia were performed before the hypoxic exposure. Also, a placebo effect could not be ruled out because of the natural hypobaric hypoxia exposure studied (Jungfraujoch Mountain). Finally, peak HR, and not HR_max_, is usually recorded in patients due to the difficulty of obtaining the “true” HR_max_. Such misinterpretation can explain these paradoxical observations. In Figure 5, patients with different diseases were merged (e.g., chronic heart failure, Fontan patients, coronary artery disease) due to the small number of studies available. To confound results further, it is unknown if the specific pathology of these patients and the pharmacological agents they take modulate the HR_max_ response with altitude. It has been recently highlighted that hypoxic exposure is safe for patients with cardiovascular diseases if specific recommendations are followed (for more details see Parati et al., 2018). However, the authors also highlight that healthy subjects taking carvedilol, a non-selective beta-blocker, display significant reductions in blood pressure responses to altitude, which was associated with reduced SaO_2_ and exercise tolerance. Whenever possible, these authors proposed ß1 selective should be used in place of non-selective beta-blockers in chronic heart failure patients. Nevertheless, experiments that involve the discontinuation of drugs that have chronotropic effects on the heart, need to be performed at altitude to adequately address this question.

### Effect of genetics

The effects of hypoxia on arterial oxygenation, VO_2max_ and HR_max_ in lowlanders depends on genetic factors (Masschelein et al., 2015). In this latter study, the authors found extremely high similarity amongst twins. The drop in HR_max_ ranged 3 and 27 bpm, but the variance within twins was approximately sevenfold less than between twins. This suggests that HR responses during exercise in acute hypoxia are dependent on genotype, but here again more research is needed.

### Cardiac function

The decrease in HR_max_ can be considered as a limiting factor of performance at altitude. However, a lower HR_max_ associated with a decreased cardiac output, could alleviate the limitation in diffusion, and thus the decrement in *S*aO_2_ and VO_2max_. Consistent with this is the greater decrease in HR_max_ in acute hypoxia in endurance athletes, who have the most important diffusion limitation (Dempsey et al., 1982). This mechanism is also protective for the heart, an organ that depends on oxygen supply too. During maximal exercise in healthy subjects, the coronary flow reserve has been shown to be limited to one-third above what is prevailing at sea level (Kaijser et al., 1990). Therefore, the compensation of decreased CaO_2_ by increasing coronary blood flow is not possible above a certain altitude. The only option to preserve cardiac integrity is thus to decrease myocardial oxygen demand and therefore HR_max_. All these changes in cardiac chronotropic activity do not seem to be associated with alterations in the inotropic function (Boussuges et al., 2000; Boushel et al., 2001). In healthy individuals, left ventricular function and myocardial oxygen supply was found to be maintained during maximal exercise at an altitude of 7,625 m, at which HR_max_ was reduced by 20% (Sutton et al., 1988).

Thus, evidence strongly suggests that in moderate acute hypoxia, HR_max_ is reduced. It should be acknowledged that large inter-study (reporting themselves large inter-subject) variability exists. But this decrement should be clearly specified, as it has been previously reported in a review paper, which focused on the use of exercise training in hypoxia that hypoxia trigger an increase in both basal and maximal HR_max_, which is contradictory to the current observation (Urdampilleta et al., 2012). However, the mechanisms leading to this HR_max_ decline remain to be identified.

## Mechanisms involved in the decrement of HR_max_ during acute hypoxia exposure

### Arterial oxygen saturation

**Figure 6:**
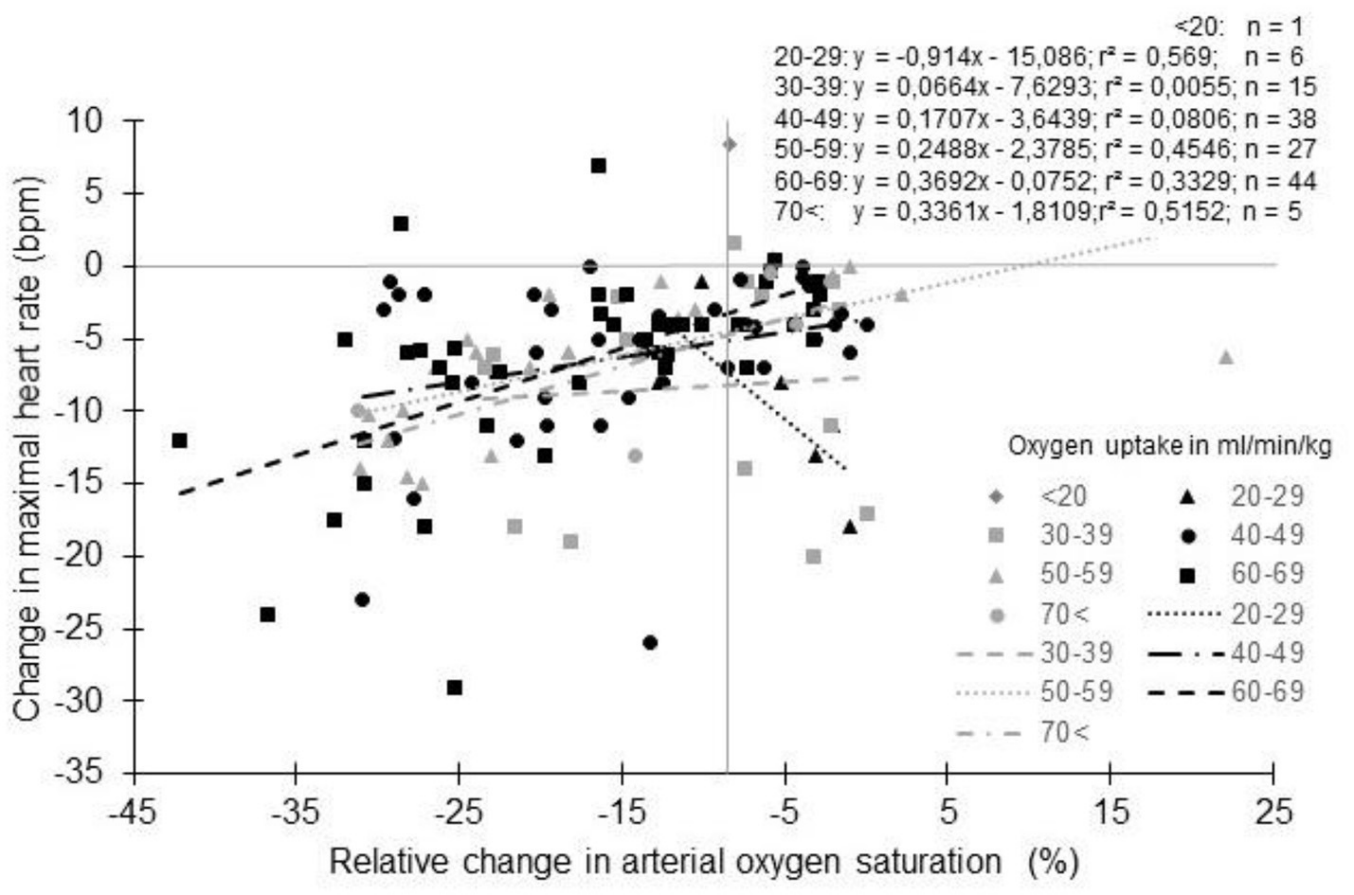
Change in maximal heart rate with altitude as a function of arterial oxygen saturation: Effect of physical fitness. Each data point represents the average value from the relevant group, as presented in previously published studies (references in Supplementary Table S1).

As reported (Supplementary Table S1, Figure 5), untrained subjects usually have a lower decrease in HR_max_ with altitude than trained subjects. The lower reduction in *S*aO_2_ generally observed in these subjects could be an explanation (Lawler et al., 1988; Martin and O'Kroy, 1993; Robergs et al., 1998; Mucci et al., 2004). During training, the repetition of long duration exercises by subjects who exhibit arterial oxygen desaturation could induce a situation similar to a chronic exposure to hypoxia. As with chronic exposure to hypoxia, chronic exercise reduces the number of cardiac ß-receptors (Werle et al., 1990). The repeated arterial oxygen desaturation with training could interfere with the effect of acute hypoxia exposure. It has been suggested that in normoxia, subjects with exercise-induced arterial hypoxemia (EIH) could present a lower HR_max_ due to the repeated exposure to exercise hypoxemia (Zavorsky, 2000). Even if not always reported (Chapman et al., 1999), subjects with EIH usually have a greater reduction in HR_max_ with a lower *S*aO_2_ than subjects without EIH (non-EIH) (Benoit et al., 2003; Grataloup et al., 2007). Also, a correlation exists between *S*aO_2_ in hypoxia and the reduction in HR_max_ (Grataloup et al., 2007). *S*aO_2_ could thus be considered as a determining factor in the HR_max_ decline with acute hypoxia even if evidence suggests that only low values of *S*aO_2_ could alter HR_max_ (Grataloup et al., 2007). In the current literature search, we found 322 groups for whom both *S*aO_2_ and HR_max_ at the end of an incremental exercise to exhaustion in normoxia and hypoxia were reported. When the change in HR_max_ was plotted vs. the percentage of change in *S*aO_2_ (Figure 6), the hypothesized trend seems to be confirmed: overall the greater the desaturation, the greater the decline in HR_max_. However, the correlation remains low (Grataloup et al., 2007), with large variability in the decrease in HR_max_ for a given decrease in *S*aO_2_, highlighting that other contributors alter HR_max_ in hypoxia.

### Myocardial electrophysiology

Potential explanations for the reduced HR_max_ include myocardial dysfunction, as suggested by Benoit et al. (2003). A direct effect of hypoxia on the cardiac electrophysiological properties including repolarization length and transmission time across the auriculo-ventricular node, occurs and relates to a reduction in HR_max_ (Roche et al., 2003; Siebenmann and Lundby, 2015). The greater hypoxemia observed in EIH subjects in the hypoxic condition could induce greater modifications of electrophysiological cardiac properties (Grataloup et al., 2007).

### Autonomic nervous system

Another contributor, likely the major one, seems to be the change in the activity of the autonomic nervous system. The reduction in HR_max_ in hypoxia has been linked to an alteration in sympathetic and parasympathetic activity mediated chronotropic function, with a densitization of the adrenergic pathway and/or an upregulation of the parasympathetic system (Hartley et al., 1974; Richalet et al., 1988, 1992; Kacimi et al., 1993; Boushel et al., 2001; Favret and Richalet, 2007). But the effect of acute hypoxia on the autonomic nervous system is not as clear (Rostrup, 1998; Naeije, 2010). Sympathetic traffic may be slightly decreased (Bouissou et al., 1986; Seals et al., 1991), unchanged (Lundby et al., 2001a) or may increase (Hughson et al., 1995) during maximal exercise in acute hypoxia. Using domperidone, a D2-receptors blocker, Lundby et al. (2001b) demonstrated that hypoxic exercise in humans activates D2-receptors with a subsequent decrease in circulating noradrenaline. However, HR_max_ was unaffected by domperidone, indicating that dopamine D2-receptors are not involved in the HR_max_ decrement (Lundby et al., 2001b). In that study, marked changes in plasma noradrenaline levels had no effect on HR_max_. This points toward a postsynaptic located mechanism. Oxygen breathing completely reversed the decrease in HR_max_ to values not different from those at sea level. This led the authors to conclude that uncoupling, and not down-regulation, of cardiac adrenoreceptors is responsible for the early decrease in HR_max_ during hypoxic exercise. However, these results were obtained after 24 h (Lundby et al., 2001b) or a few days of acclimatization (Kacimi et al., 1992). During acute hypoxia, the involvement of the autonomic nervous system requires further study. Although the previous changes in receptor function could be rapidly triggered by exposure to hypoxia and high adrenergic activity, it is unlikely that these mechanisms are involved in a short exposure of only a few minutes/hours. It has been hypothesized that an increase in parasympathetic activity, rather than decreased sympathetic activity, might be responsible for the blunting of the cardiac chronotropic function during acute hypoxia (Lundby et al., 2001a). Indeed, HR_max_ can be partly restored toward sea level values by administration of atropine, a parasympathetic antagonist (Hartley et al., 1974; Bouschel et al., 1999; Bogaard et al., 2002). Moreover, previous studies have concluded that plasma norepinephrine tends to underestimate sympathetic traffic during acute hypoxia (Escourrou et al., 1984), due to increased reuptake of norepinephrine compared to normoxia (Leuenberger et al., 1991). Therefore, it seems unlikely that the decreased HR_max_ in acute hypoxia is related to a decrease in sympathetic activity (Naeije, 2010). Mechanistically, cardiac output can be blunted by direct hypoxic excitation of medullary neurons, which project to cardiovagal neurons, causing bradycardia (Golanov et al., 2000). Taken together, these results suggest that both sympathetic and parasympathetic activity is increased during maximal aerobic exercise in acute hypoxia. When the two efferent arms of the autonomic nervous system are activated, the parasympathetic activity generally dominates at the heart level (Levy, 1971; Mendelowitz, 1999), in accordance with the decrease in resting HR observed during cold water immersion despite a large increase in catecholamines in healthy subjects (Mourot et al., 2007). This could be a valuable explanation of the HR_max_ decline with increasing acute hypoxia levels. Another explanation of the reduced HR_max_ could be that hypoxia reduced myocardial contractility and/or the rate of diastolic filling (Shephard et al., 1988), but this remains to be confirmed (Boussuges et al., 2000; Boushel et al., 2001).

### Central factors

Since the peak power output achieved is also reduced in hypoxia, an alternative hypothesis could be that HR_max_ is lowered due to a decrease in exercise effort. However, maximal plasma lactate and catecholamine concentrations (Stenberg et al., 1966; Bouissou et al., 1986; Lundby et al., 2000, 2001a) as well as perceptual responses (Horstman et al., 1979) at the termination of exercise are often similar at altitude and sea level. Maximal skeletal muscle VO_2_ could also dictate maximal cardiac output in hypoxia (Wagner, 2000). Arterial hypoxemia may induce a decrease in muscle VO_2max_, which in turn determines a decrease in cardiac output. This is in agreement with the association between the change in HR_max_ and the change in VO_2max_ found in many studies (Peltonen et al., 2001). A reduced central drive to the cardioacceleratory centers could also be suggested. Chronic (Kayser et al., 1994) and acute hypoxia (Peltonen et al., 1995) do not alter neuromuscular fatigue, and hypoxia-induced systemic adaptations are not triggered by neural feedback from working muscles (Kjaer et al., 1999). This eliminates a potential peripheral effect. Most probably, a decrease in oxygen content is perceived by the central nervous system. Otherwise, the increase in cerebrovascular blood flow (Harik et al., 1996) would be a unsuitable adaptation. Two studies support the notion of a central nervous system limitation to exercise performance in hypoxia equivalent to 5,300 m (Calbet et al., 2003a,b). During an incremental test in normoxia, task failure is most likely caused by peripheral mechanisms while central mechanisms seem to prevail in severe hypoxia (Calbet et al., 2003a; Subudhi et al., 2007). These explanations are in accordance with the hypothesis of a “central governor” that regulates the mass of skeletal muscle recruited during exercise and protects the heart from ischemia (Noakes et al., 2001). With, as a consequence, a decreased in HR_max_. This is agreement with the fact that the changes in maximal power and maximal cardiac output are very similar (Peltonen et al., 2001). Noakes' hypothesis is that the oxygen tension in the coronary vascular bed is the monitored variable by the “central governor” to prevent a progressive myocardial ischemia (Noakes et al., 2001). However, this hypothesis has certain limitations. The results of previous studies suggest that the signals that inhibit the increase of HR in acute hypoxia may arise somewhere else than in the myocardium itself, likely the central nervous system (Kayser et al., 1994; Harik et al., 1996; Peltonen et al., 1997; Kjaer et al., 1999; Calbet et al., 2003a,b). Also, the fact that the drop in HR_max_ appears at low altitude (below or at 1,000 m; Gore et al., 1996, 1997; Robergs et al., 1997) indicates that it is improbable that such a low altitude would constitute a significant challenge for the central nervous system.

The inter-individual variability in the decrease in HR_max_ with the hypoxic stimulus is important and the underlying mechanisms still needs to be specified. Nevertheless, this decrease has practical effects that should be taken into consideration, especially when prescribing hypoxic exercise for patients.

## Consequences in clinical practice

As seen in Figure 1, an increasing number of research studies focuses on exercise training in hypoxia for patients, with authors strongly arguing about its potential superior effects compared to exercise in normoxia (Wee and Climstein, 2015; Millet et al., 2016b; Hobbins et al., 2017; Thiersch et al., 2017). In hypoxia, the prescription of exercise intensity requires considerable attention due to the reduction in aerobic capacity (Friedmann et al., 2005). Hence, a proper evaluation of the superiority of a hypoxic training program should be done to decide whether or not a patient should be involved in this type of exercise environment. While dealing with hypoxic training to increase an athlete's performance, it is highly recommended to strictly control potential bias such as the placebo effect and to compare similar training programs to ensure that results obtained are due solely to hypoxia (Lundby et al., 2012).

### Use of HR for exercise prescription

In studies which compare the hypoxia and normoxia results in patients, prescription of the intensity of an aerobic-based exercise training program is often based on percentage of HR_max_ (Netzer et al., 2008; Kong et al., 2014; Park and Lim, 2017) or HRR (Greie et al., 2006; Mao et al., 2011; Schreuder et al., 2014; Gutwenger et al., 2015) (Supplementary Table S2), in agreement with guidelines (Pescatello et al., 2004, 2015). Using this approach, studies involving patients and healthy subjects that used a percentage of theoretical or normoxic HR_max_/HRR have found additional effects of hypoxic vs. normoxic training (Supplementary Table S2). Hendriksen and Meeuwsen, 2003) compared training at 60-70% of HRR at sea level and 2,500 m and found that intermittent hypobaric training can improve the aerobic and anaerobic energy supplying system. Kong et al. (2014) showed that hypoxic training between 2,000 and 3,000 m at 60–70% of HR_max_ caused more weight loss than normoxic training in obese young adults. Wiesner et al. (2010) reported a better response in physical fitness, metabolic risk markers, and body composition in obese subjects after comparing training completed at the same absolute HR (HR at 65% of VO_2max_ in normoxia). The absolute HR were identical in both normoxic and hypoxic groups in the study of Bailey et al. (2000) who reported an additive cardioprotective effect of normobaric hypoxia over normoxic training. This apparent superiority has led authors of reviews dealing with exercise training in hypoxia to suggest using specific percentages of HR_max_ (e.g., 55-85% HR_max_ for Wee and Climstein, 2015, and 60–70% of HR_max_ for Hobbins et al., 2017). Unfortunately, they did not specify whether the HR_max_ was the normoxic or hypoxic one. Since HR_max_ decreases with altitude, the distinction between normoxic and hypoxic HR_max_ is important and has several practical implications.

The altitude commonly used for exercise training with patients is often 2,000–3,500 m; (Haufe et al., 2008; Netzer et al., 2008; Wiesner et al., 2010; Kong et al., 2014; Schreuder et al., 2014; Gutwenger et al., 2015; Park and Lim, 2017; Pramsohler et al., 2017). As highlighted elsewhere (Schreuder et al., 2014), for this altitude range VO_2max_ under hypoxia is approximately 91% of the value achieved under normoxia. It means that HR_max_ under hypoxia is reached at 91% of maximal VO_2_-uptake at sea level. Therefore, training at 70–75% of the HRR under hypoxia corresponds with an absolute level of oxygen uptake that is already achieved at 64–68% of the HRR when exercise is performed under hypoxia. Hypoxia during exercise shifts ventilation, HR and blood lactate concentration vs. intensity curves to the left compared with sea-level values (Friedmann et al., 2004). In addition, the blood lactate concentration vs. HR curve shifts to the left, especially at high HR and, consequently, training HR may be decreased during training in hypoxia (Levine and Stray-Gundersen, 1997; Peltonen et al., 2001).

### Magnitude of the effect of hypoxia

Based on Figure 2, the decrease of HR_max_ could be expected to be 5 and 7.5 bpm at 2,500 and 3,500 m, respectively. If we consider the author of this manuscript (40 years-old and a HR_max_ at sea level of 181 bpm), an overestimation of 3 bpm (2.8%) and 5 bpm (2.8%) of target HR for moderate (40% HR_max_) and vigorous (90% HR_max_) intensity exercise, respectively, would have been observed for an exercise training session at 2,500 m. At 3,000 m, it would have been 4 bpm (3.5%) and 6 bpm (3.5%), respectively. Focusing only on the altitude gain ranging from 2,500 to 3,500 m (Supplementary Table S1), the average HR_max_ decrement is 4.2 ± 3.9 bpm. In that case, the overestimation is 3 bpm (2.3%) and 4 bpm (2.3%) for moderate and vigorous exercise, respectively. Also, resting HR increases under hypoxia due to increase sympathetic and decrease parasympathetic activity (Windsor et al., 2010; Siebenmann and Lundby, 2015). This increase could reach 4-5 bpm (Friedmann et al., 2004; Subudhi et al., 2011; Horiuchi et al., 2017), even at low altitude (equivalent to only 1,650 m for (Subudhi et al., 2011)). Knowing the resting HR of the author (55 bpm), the overestimation could have been 2 bpm (1.9%) for moderate (40% HRR) and 4 bpm (2.6%) for vigorous (85% HRR) intensity exercise at 2,500 m. The corresponding values would have been 2 bpm (2.4%) and 5 bpm (3.3%) at 3,500 m. Finally, by using the average decrement of 4.2 ± 3.9 bpm of HR_max_, the overestimation would have been 1 bpm (1.6%) and 3 bpm (2.2%) for moderate and vigorous intensity calculated from HRR. Using the data presented in Figure 5 for VO2_max_ group 50-59 ml/min/kg, the overestimation is roughly the same for each given example. However, using the data presented in Figure 4 for 30–39 years age group, the overestimation increases by 1% in each example that has been provided. The overestimation calculated in relation to this particular case is consistent with those calculated from the data obtained in healthy subjects in previous studies (Friedmann et al., 2004; Horiuchi et al., 2017; Subudhi et al., 2011). Hence, depending on the calculation, a systematic overestimation of the intensity by 1–4% would be obtained in hypoxia. Fortunately, in this specific case, HR_max_ is very close to the predicted HR_max_ indirectly derived from the age. But such derived HR_max_ could lead to large error in the prediction (Robergs, and Landwher, 2002; Gallagher et al., 2015). At the group level with the data obtained in Supplementary Table S1, the calculated 220-age HR_max_ was 7.8 ± 9.1 bpm higher than the measured HR_max_ in normoxia, and 14.3 ± 10 bpm higher in hypoxia. Using hypobaric or normobaric hypoxia could also be of importance, since a significantly higher resting HR in hypobaric hypoxia compared to normobaric hypoxia have been reported (Savourey et al., 2003). The importance of such difference as well as the underlying mechanisms has to be specified (Beidleman et al., 2014; Richard et al., 2014; Saugy et al., 2016; Bourdillon et al., 2017; Woods et al., 2017).

### Practical consequences of the overestimation

In normoxia, the intraclass correlation coefficients (ICC) for submaximal and maximal HR could be considered as high, since they have been reported to be greater than 0.9, the higher the HR, the lower the variation (Lamberts et al., 2004; Tulumen et al., 2011). But expressed in bpm, the day-to-day variations in submaximal HR could be estimated to around 5-8 bpm (Lamberts et al., 2004). It means that the difference in HR_max_ between normoxia and hypoxia that could be expected at 2,500 m (Figure 2 and specific example given above) could be hidden by the spontaneous fluctuations of HR for a given training load. Hence, this difference could be considered as being of low importance. But because there is actually no reason to consider that the day-to-day fluctuation of HR in hypoxia would be different from the variation in normoxia, it remains that hypoxia leads to a decrease in HR_max_. Such overestimation, even if of moderate magnitude, could lead to the prescription of exercise intensities difficult to reach or even unachievable by the participant. It may result in decreased adherence to exercise training sessions (Perri et al., 2002). Alternatively, in these studies the hypoxic groups would have exercised with higher intensities than the control group and that studies comparing the effect of exercise training in hypoxia to normoxia may not be simply comparing a difference in the inspired oxygen fraction, but also a difference in (relative) exercise intensity. Since this favors a systematic higher exercise intensity in hypoxia, it casts doubt on the validity of the previously reported results. Training at higher intensities than sea-level could trigger a state of fatigue. Even without the overestimation, the risk of overloading exists in hypoxia. As an example, Debevec et al. (2010) matched 20 training sessions at an HR level corresponding to that elicited at 50% of peak power output attained in normoxia or hypoxia, respectively. The group who trained in hypoxia experienced a significant decrease in HR_max_ during the normoxic incremental test to exhaustion post-training. They suspected a possible state of fatigue for this group and highlighted the need pay attention to the sum of exercise and the hypoxic stimulus (Ventura et al., 2003; Friedmann et al., 2005). Using HR_max_ at sea level or predicted from age during physiological assessment could also affect the conclusions of physiological studies. For example, HR relative to 220-age as an index of the intensity of exercise was used to understand the effect of altitude on microvascular parameters in both environments, thus overestimating the exercise intensity in hypoxia (Bauer et al., 2006). On the other hand, as no real adverse effects have been reported for this range of exercise intensity/altitude, this overestimation underlined that hypoxic exercise training seems well tolerated by patients (Agostoni, 2013; Levine, 2015; Parati et al., 2018) and provides a certain level of flexibility for the execution of the training program.

## Conclusions and future perspectives

A body of scientific literature exists on the evaluation of passive exposure and/or exercise training in altitude/hypoxic environment in healthy subjects. Despite this, debates still exists on the benefits and physiological effects of hypoxic training. Nevertheless, the use of exercise training in hypoxia has grown in popularity with patients. Studies have provided encouraging results, but the effectiveness and potential superiority of hypoxic training over normoxic training remains to be established. To achieve this, the exercise training programs performed by the experimental and control groups should be comparable to allow a rigorous evaluation of the isolated hypoxic stimulus. The best metric to quantify the exercise training load needs to be specified, but the prescription of exercise for patients largely rely on HR recordings. This review underlines that HR_max_ decreases progressively with increasing hypoxia. The dose–response is roughly linear and starts at a low altitude. However, the literature reports large inter-study and inter-individual variabilities. Since the studies are usually conducted with a limited number of participants, this hampers findings of significant difference compared to sea-level especially at low altitudes where the change is limited. Sex and age do not seem to be major contributors to the HR_max_ decrement with altitude. Rather, it seems that the greater the reduction in *S*aO_2_, the greater the reduction in HR_max_, likely due to an over activity of the parasympathetic nervous system. Only a few studies reported HR_max_ at sea/low level and altitude with patients. Together with very different study designs, it is difficult to draw firm conclusions amongst these specific populations. The magnitude of the decrease in HR_max_ seems to be augmented after days at high altitude (acclimatization) and therefore also depends on the duration of hypoxic exposure. It is not known if over the course of a training program HR_max_ decreases in the same way, even if unlikely (a huge difference between daily exposure on one side and 3-5 sessions of 30-90 min per week exists in terms of hypoxic exposure). Hence, much remains to be done in this field and forthcoming studies with appropriate designs need to be conducted to answer questions in both the physiological and clinical area. In the first category, the effects of hypoxia on HR_max_ (but also on resting HR which appears of importance for prescription based on HRR) amongst specific populations have to be better addressed, with a specific focus on sex and age. Also, it is of importance to better assess these effects in specific pathologies (in particular cardiovascular and metabolic diseases), as well as determining potential interaction with drugs. The difference between normobaric and hypobaric hypoxia should also be addressed. In all of these situations, the specific impact of *S*aO_2_ and the autonomic nervous system should be taken into consideration. In the second category, a thorough evaluation of the potential superiority of exercise training in hypoxia over normoxia on relevant outcomes need to be done with a well-controlled design that involves accounting for the placebo effect and an adequate prescription of exercise training intensity based on heart rate. Under conditions of hypoxic exercise training, the long-term effect of hypoxia exposure on HR_max_ and the potential need to adjust the relative HR target should also be addressed as well as the need to account for chronotropic effects of drugs. In some years, a review similar to the present one would be very instructive.

## Author contributions

LM: Lead author, study writing. The manuscript presents clearly results, honestly, and without fabrication, falsification, or inappropriate data manipulation. Results of the present study do not constitute endorsement by ACSM.

### Conflict of interest statement

The author declares that the research was conducted in the absence of any commercial or financial relationships that could be construed as a potential conflict of interest.

## References

[B1] Achten J. Jeukendrup A. E. (2003). Heart rate monitoring. Sports Med. 33, 517–538. 10.2165/00007256-200333070-00004 12762827

[B2] Agostoni P. (2013). Considerations on safety and treatment of patients with chronic heart failure at high altitude. High Alt. Med. Biol. 14, 96–100. 10.1089/ham.2012.1117 23795728

[B3] Agostoni P. Cattadori G. Guazzi M. Bussotti M. Conca C. Lomanto M.. (2000). Effects of simulated altitude-induced hypoxia on exercise capacity in patients with chronic heart failure. Am. J. Med. 109, 450–455. 10.1016/S0002-9343(00)00532-5 11042233

[B4] Angermann M. Hoppeler H. Wittwer M. Däpp C. Howald H. Vogt M. (2006). Effect of acute hypoxia on maximal oxygen uptake and maximal performance during leg and upper-body exercise in nordic combined skiers. Int. J. Sports Med. 27, 301–306. 10.1055/s-2005-865652 16572373

[B5] Bailey D. M. Davies B. Baker J. (2000). Training in hypoxia: modulation of metabolic and cardiovascular risk factors in men. Med. Sci. Sports Exerc. 32, 1058–1066. 10.1097/00005768-200006000-00004 10862530

[B6] Bailey D. M. Davies B. Young I. S. (2001). Intermittent hypoxic training: implications for lipid peroxidation induced by acute normoxic exercise in active men. Clin. Sci. Lond. Engl. 101, 465–475. 10.1042/cs1010465 11672451

[B7] Bärtsch P. Saltin B. (2008). General introduction to altitude adaptation and mountain sickness. Scand. J. Med. Sci. Sports 18(Suppl. 1), 1–10. 10.1111/j.1600-0838.2008.00827.x 18665947

[B8] Bauer A. Demetz F. Bruegger D. Schmoelz M. Schroepfer S. Martignoni A.. (2006). Effect of high altitude and exercise on microvascular parameters in acclimatized subjects. Clin. Sci. Lond. Engl. 110, 207–215. 10.1042/CS20050217 16194151

[B9] Beidleman B. A. Fulco C. S. Staab J. E. Andrew S. P. Muza S. R. (2014). Cycling performance decrement is greater in hypobaric versus normobaric hypoxia. Extreme Physiol. Med. 3:8. 10.1186/2046-7648-3-8 24778792 PMC4002198

[B10] Benoit H. Busso T. Castells J. Geyssant A. Denis C. (2003). Decrease in peak heart rate with acute hypoxia in relation to sea level VO(2max). Eur. J. Appl. Physiol. 90, 514–519. 10.1007/s00421-003-0899-y 12898267

[B11] Benoit H. Busso T. Prieur F. Castells J. Freyssenet D. Lacour J. R.. (1997). Oxygen uptake during submaximal incremental and constant work load exercises in hypoxia. Int. J. Sports Med. 18, 101–105. 10.1055/s-2007-972603 9081265

[B12] Billat V. L. Lepretre P. M. Heubert R. P. Koralsztein J. P. Gazeau F. P. (2003). Influence of acute moderate hypoxia on time to exhaustion at vVO2max in unacclimatized runners. Int. J. Sports Med. 24, 9–14. 10.1055/s-2003-37251 12582946

[B13] Bogaard H. J. Hopkins S. R. Yamaya Y. Niizeki K. Ziegler M. G. Wagner P. D. (2002). Role of the autonomic nervous system in the reduced maximal cardiac output at altitude. J. Appl. Physiol. 93, 271–279. 10.1152/japplphysiol.00323.2001 12070214

[B14] Bonetti D. L. Hopkins W. G. (2009). Sea-level exercise performance following adaptation to hypoxia: a meta-analysis. Sports Med. Auckl. NZ 39, 107–127. 10.2165/00007256-200939020-00002 19203133

[B15] Bouissou P. Péronnet F. Brisson G. Hélie R. Ledoux M. (1986). Metabolic and endocrine responses to graded exercise under acute hypoxia. Eur. J. Appl. Physiol. 55, 290–294. 10.1007/BF02343801 3525153

[B16] Bourdillon N. Saugy J. Schmitt L. Rupp T. Yazdani S. Vesin J. M.. (2017). Acute and chronic changes in baroreflex sensitivity in hypobaric vs. normobaric hypoxia. Eur. J. Appl. Physiol. 117, 2401–2407. 10.1007/s00421-017-3726-6 28956166

[B17] Bouschel R. Calbet J. A. Radegran G. Sondergaard H. Wagner H. Saltin B. (1999). Parasympathetic neural tone induces the lowering of exercise heart rate in hypoxia, but blocking of the vagal nerve has no influence on O2 transport and exercise performance. FASEB J. 13:LB56.

[B18] Boushel R. Calbet J. A. Rådegran G. Sondergaard H. Wagner P. D. Saltin B. (2001). Parasympathetic neural activity accounts for the lowering of exercise heart rate at high altitude. Circulation 104, 1785–1791. 10.1161/hc4001.097040 11591615

[B19] Boussuges A. Molenat F. Burnet H. Cauchy E. Gardette B. Sainty J. M. (2000). Operation Everest III (Comex'97): modifications of cardiac function secondary to altitude-induced hypoxia. An echocardiographic and Doppler study. Am. J. Respir. Crit. Care Med. 161, 264–270. 10.1164/ajrccm.161.1.9902096 10619830

[B20] Brocherie F. Girard O. Faiss R. Millet G. P. (2017). Effects of repeated-sprint training in hypoxia on sea-level performance: a meta-analysis. Sports Med. Auckl. NZ 47, 1651–1660. 10.1007/s40279-017-0685-3 28194720

[B21] Brothers M. D. Hilger K. Carson J. M. Sullivan L. Byrnes W. C. (2007). GXT responses in altitude-acclimatized cyclists during sea-level simulation. Med. Sci. Sports Exerc. 39, 1727–1735. 10.1249/mss.0b013e3181238a3f 17909399

[B22] Calbet J. A. Boushel R. Rådegran G. Søndergaard H. Wagner P. D. Saltin B. (2003a). Determinants of maximal oxygen uptake in severe acute hypoxia. Am. J. Physiol. Regul. Integr. Comp. Physiol. 284, R291–R303. 10.1152/ajpregu.00155.2002 12388461

[B23] Calbet J. A. L. Boushel R. Radegran G. Sondergaard H. Wagner P. D. Saltin B. (2003b). Why is VO2 max after altitude acclimatization still reduced despite normalization of arterial O2 content? Am. J. Physiol. Regul. Integr. Comp. Physiol. 284, R304–316. 10.1152/ajpregu.00156.2002 12388462

[B24] Casillas J. M. Gudjoncik A. Gremeaux V. Aulagne J. Besson D. Laroche D. (2017). Assessment tools for personalizing training intensity during cardiac rehabilitation: Literature review and practical proposals. Ann. Phys. Rehabil. Med. 60, 43–49. 10.1016/j.rehab.2016.01.011 26996956

[B25] Cerretelli P. (1976). Limiting factors to oxygen transport on Mount Everest. J. Appl. Physiol. 40, 658–667. 10.1152/jappl.1976.40.5.658 931890

[B26] Cerretelli P. Bordoni U. Debijadij R. Saracino F. (1967). Respiratory and circulatory factors affecting the maximal aerobic power in hypoxia. Arch. Fisiol. 65, 344–357. 5612425

[B27] Chapman R. F. Emery M. Stager J. M. (1999). Degree of arterial desaturation in normoxia influences VO2max decline in mild hypoxia. Med. Sci. Sports Exerc. 31, 658–663. 10.1097/00005768-199905000-00006 10331884

[B28] Christensen E. H. Forbes W. H. (1937). Der Kreislauf in großen Höhen. Skand. Arch. Für Physiol. 76, 75–87. 10.1111/j.1748-1716.1937.tb01584.x

[B29] Clark S. A. Bourdon P. C. Schmidt W. Singh B. Cable G. Onus K. J.. (2007). The effect of acute simulated moderate altitude on power, performance and pacing strategies in well-trained cyclists. Eur. J. Appl. Physiol. 102, 45–55. 10.1007/s00421-007-0554-0 17882451

[B30] Cymerman A. Reeves J. T. Sutton J. R. Rock P. B. Groves B. M. Malconian M. K.. (1989). Operation Everest II: maximal oxygen uptake at extreme altitude. J. Appl. Physiol. 66, 2446–2453. 10.1152/jappl.1989.66.5.2446 2745305

[B31] Davison G. W. Morgan R. M. Hiscock N. Garcia J. M. Grace F. Boisseau N.. (2006). Manipulation of systemic oxygen flux by acute exercise and normobaric hypoxia: implications for reactive oxygen species generation. Clin. Sci. (Lond). 110, 133–141. 10.1042/CS20050135 16197367

[B32] Debevec T. Amon M. Keramidas M. E. Kounalakis S. N. Pisot R. Mekjavic I. B. (2010). Normoxic and hypoxic performance following 4 weeks of normobaric hypoxic training. Aviat. Space Environ. Med. 81, 387–393. 10.3357/ASEM.2660.2010 20377142

[B33] Dekerle J. Mucci P. Carter H. (2012). Influence of moderate hypoxia on tolerance to high-intensity exercise. Eur. J. Appl. Physiol. 112, 327–335. 10.1007/s00421-011-1979-z 21556815

[B34] Dempsey J. Hanson P. Pegelow D. Claremont A. Rankin J. (1982). Limitations to exercise capacity and endurance: pulmonary system. Can. J. Appl. Sport Sci. J. Can. Sci. Appl. Au Sport 7, 4–13. 6807559

[B35] De Pauw K. Roelands B. Cheung S. S. de Geus B. Rietjens G. Meeusen R. (2013). Guidelines to classify subject groups in sport-science research. Int. J. Sports Physiol. Perform. 8, 111–122. 10.1123/ijspp.8.2.111 23428482

[B36] Díaz-Gutiérrez J. Martínez-González M. Á. Pons Izquierdo J. J. González-Muniesa P. Martínez J. A. Bes-Rastrollo M. (2016). Living at higher altitude and incidence of overweight/obesity: prospective analysis of the SUN cohort. PLoS ONE 11:e0164483. 10.1371/journal.pone.0164483 27812092 PMC5094724

[B37] Dill D. B. Myhre G. Phillips E. E. Brown D. K. (1966). Work capacity in acute exposures to altitude. J. Appl. Physiol. 21, 1168–1176. 10.1152/jappl.1966.21.4.1168 5916646

[B38] Drinkwater B. L. Folinsbee L. J. Bedi J. F. Plowman S. A. Loucks A. B. Horvath S. M. (1979). Response of women mountaineers to maximal exercise during hypoxia. Aviat. Space Environ. Med. 50, 657–662. 486011

[B39] Dufour S. P. Ponsot E. Zoll J. Doutreleau S. Lonsdorfer-Wolf E. Geny B.. (2006). Exercise training in normobaric hypoxia in endurance runners. I. Improvement in aerobic performance capacity. J. Appl. Physiol. 100, 1238–1248. 10.1152/japplphysiol.00742.2005 16540709

[B40] Ekblom B. Huot R. Stein E. M. Thorstensson A. T. (1975). Effect of changes in arterial oxygen content on circulation and physical performance. J. Appl. Physiol. 39, 71–75. 10.1152/jappl.1975.39.1.71 1150596

[B41] Erdmann J. Sun K. T. Masar P. Niederhauser H. (1998). Effects of exposure to altitude on men with coronary artery disease and impaired left ventricular function. Am. J. Cardiol. 81, 266–270. 10.1016/S0002-9149(97)00901-6 9468065

[B42] Escourrou P. Johnson D. G. Rowell L. B. (1984). Hypoxemia increases plasma catecholamine concentrations in exercising humans. J. Appl. Physiol. 57, 1507–1511. 10.1152/jappl.1984.57.5.1507 6520045

[B43] Fagraeus L. Karlsson J. Linnarsson D. Saltin B. (1973). Oxygen uptake during maximal work at lowered and raised ambient air pressures. Acta Physiol. Scand. 87, 411–421. 10.1111/j.1748-1716.1973.tb05405.x 4697153

[B44] Faiss R. von Orelli C. Dériaz O. Millet G. P. (2014). Responses to exercise in normobaric hypoxia: comparison of elite and recreational ski mountaineers. Int. J. Sports Physiol. Perform. 9, 978–984. 10.1123/ijspp.2013-0524 24664934

[B45] Faiss R. Willis S. Born D. P. Sperlich B. Vesin J. M. Holmberg H. C.. (2015). Repeated double-poling sprint training in hypoxia by competitive cross-country skiers. Med. Sci. Sports Exerc. 47, 809–817. 10.1249/MSS.0000000000000464 25083727

[B46] Favret F. Richalet J. P. (2007). Exercise and hypoxia: the role of the autonomic nervous system. Respir. Physiol. Neurobiol. 158, 280–286. 10.1016/j.resp.2007.04.001 17521971

[B47] Feriche B. García-Ramos A. Morales-Artacho A. J. Padial P. (2017). Resistance training using different hypoxic training strategies: a basis for hypertrophy and muscle power development. Sports Med. 3:12. 10.1186/s40798-017-0078-z 28315193 PMC5357242

[B48] Ferretti G. Moia C. Thomet J. M. Kayser B. (1997). The decrease of maximal oxygen consumption during hypoxia in man: a mirror image of the oxygen equilibrium curve. J. Physiol. 498(Pt 1), 231–237. 10.1113/jphysiol.1997.sp021854 9023781 PMC1159247

[B49] Friedmann B. Bauer T. Menold E. Bärtsch P. (2004). Exercise with the intensity of the individual anaerobic threshold in acute hypoxia. Med. Sci. Sports Exerc. 36, 1737–1742. 10.1249/01.MSS.0000142307.62181.37 15595295

[B50] Friedmann B. Frese F. Menold E. Bärtsch P. (2005). Individual variation in the reduction of heart rate and performance at lactate thresholds in acute normobaric hypoxia. Int. J. Sports Med. 26, 531–536. 10.1055/s-2004-821326 16195985

[B51] Fukuda T. Maegawa T. Matsumoto A. Komatsu Y. Nakajima T. Nagai R.. (2010). Effects of acute hypoxia at moderate altitude on stroke volume and cardiac output during exercise. Int. Heart. J. 51, 170–175. 10.1536/ihj.51.170 20558906

[B52] Gallagher C. A. Willems M. E. Lewis M. P. Myers S. D. (2015). The application of maximal heart rate predictive equations in hypoxic conditions. Eur. J. Appl. Physiol. 115, 277–284. 10.1007/s00421-014-3007-6 25294663

[B53] Garcia J. A. McMinn S. B. Zuckerman J. H. Fixler D. E. Levine B. D. (1999). The role of the right ventricle during hypobaric hypoxic exercise: insights from patients after the Fontan operation. Med. Sci. Sports Exerc. 31, 269–276. 10.1097/00005768-199902000-00011 10063817

[B54] Garvican-Lewis L. A. Sharpe K. Gore C. J. (2016). Time for a new metric for hypoxic dose? J. Appl. Physiol. 121, 352–355. 10.1152/japplphysiol.00579.2015 26917695

[B55] Gatterer H. Haacke S. Burtscher M. Faulhaber M. Melmer A. Ebenbichler C.. (2015). Normobaric intermittent hypoxia over 8 months does not reduce body weight and metabolic risk factors–a randomized, single blind, placebo-controlled study in normobaric hypoxia and normobaric sham hypoxia. Obes. Facts 8, 200–209. 10.1159/000431157 26008855 PMC5644878

[B56] Girard O. Brocherie F. Millet G. P. (2017). Effects of altitude/hypoxia on single- and multiple-sprint performance: a comprehensive review. Sports Med. Auckl. NZ 47, 1931–1949. 10.1007/s40279-017-0733-z 28451905

[B57] Golanov E. V. Ruggiero D. A. Reis D. J. (2000). A brainstem area mediating cerebrovascular and EEG responses to hypoxic excitation of rostral ventrolateral medulla in rat. J. Physiol. 529(Pt 2), 413–429. 10.1111/j.1469-7793.2000.00413.x 11101651 PMC2270200

[B58] Gore C. J. Hahn A. G. Scroop G. C. Watson D. B. Norton K. I. Wood R. J.. (1996). Increased arterial desaturation in trained cyclists during maximal exercise at 580 m altitude. J. Appl. Physiol. (1985) 80, 2204–2210. 10.1152/jappl.1996.80.6.2204 8806931

[B59] Gore C. J. Little S. C. Hahn A. G. Scroop G. C. Norton K. I. Bourdon P. C.. (1997). Reduced performance of male and female athletes at 580 m altitude. Eur. J. Appl. Physiol. 75, 136–143. 10.1007/s004210050138 9118979

[B60] Grataloup O. Busso T. Castells J. Denis C. Benoit H. (2007). Evidence of decrease in peak heart rate in acute hypoxia: effect of exercise-induced arterial hypoxemia. Int. J. Sports Med. 28, 181–185. 10.1055/s-2006-924216 17111315

[B61] Greie S. Humpeler E. Gunga H. C. Koralewski E. Klingler A. Mittermayr M.. (2006). Improvement of metabolic syndrome markers through altitude specific hiking vacations. J. Endocrinol. Invest. 29, 497–504. 10.1007/BF03344138 16840826

[B62] Gutwenger I. Hofer G. Gutwenger A. K. Sandri M. Wiedermann C. J. (2015). Pilot study on the effects of a 2-week hiking vacation at moderate versus low altitude on plasma parameters of carbohydrate and lipid metabolism in patients with metabolic syndrome. BMC Res. Notes 8:103. 10.1186/s13104-015-1066-3 25885799 PMC4383206

[B63] Hamlin M. J. Lizamore C. A. Hopkins W. G. (2018). The effect of natural or simulated altitude training on high-intensity intermittent running performance in team-sport athletes: a meta-analysis. Sports Med. 48, 431–446. 10.1007/s40279-017-0809-9 29129021

[B64] Hamlin M. J. Marshall H. C. Hellemans J. Ainslie P. N. Anglem N. (2010). Effect of intermittent hypoxic training on 20 km time trial and 30 s anaerobic performance. Scand. J. Med. Sci. Sports 20, 651–661. 10.1111/j.1600-0838.2009.00946.x 19793215

[B65] Harik N. Harik S. I. Kuo N. T. Sakai K. Przybylski R. J. LaManna J. C. (1996). Time-course and reversibility of the hypoxia-induced alterations in cerebral vascularity and cerebral capillary glucose transporter density. Brain Res. 737, 335–338. 10.1016/0006-8993(96)00965-1 8930387

[B66] Hartley L. H. Vogel J. A. Cruz J. C. (1974). Reduction of maximal exercise heart rate at altitude and its reversal with atropine. J. Appl. Physiol. 36, 362–365. 10.1152/jappl.1974.36.3.362 4814308

[B67] Haufe S. Wiesner S. Engeli S. Luft F. C. Jordan J. (2008). Influences of normobaric hypoxia training on metabolic risk markers in human subjects. Med. Sci. Sports Exerc. 40, 1939–1944. 10.1249/MSS.0b013e31817f1988 18845972

[B68] Hendriksen I. J. Meeuwsen T. (2003). The effect of intermittent training in hypobaric hypoxia on sea-level exercise: a cross-over study in humans. Eur. J. Appl. Physiol. 88, 396–403. 10.1007/s00421-002-0708-z 12527969

[B69] Heubert R. A. P. Quaresima V. Laffite L. P. Koralsztein J. P. Billat V. L. (2005). Acute moderate hypoxia affects the oxygen desaturation and the performance but not the oxygen uptake response. Int. J. Sports Med. 26, 542–551. 10.1055/s-2004-821329 16195987

[B70] Hobbins L. Hunter S. Gaoua N. Girard O. (2017). Normobaric hypoxic conditioning to maximize weight loss and ameliorate cardio-metabolic health in obese populations: a systematic review. Am. J. Physiol. Regul. Integr. Comp. Physiol. 313, R251–R264. 10.1152/ajpregu.00160.2017 28679682

[B71] Hogan M. C. Cox R. H. Welch H. G. (1983). Lactate accumulation during incremental exercise with varied inspired oxygen fractions. J. Appl. Physiol. 55, 1134–1140. 10.1152/jappl.1983.55.4.1134 6629944

[B72] Hoppeler H. Klossner S. Vogt M. (2008). Training in hypoxia and its effects on skeletal muscle tissue. Scand. J. Med. Sci. Sports 18(Suppl. 1), 38–49. 10.1111/j.1600-0838.2008.00831.x 18665951

[B73] Horiuchi M. Endo J. Dobashi S. Handa Y. Kiuchi M. Koyama K. (2017). Muscle oxygenation profiles between active and inactive muscles with nitrate supplementation under hypoxic exercise. Physiol. Rep. 5:e13475. 10.14814/phy2.13475 29066597 PMC5661236

[B74] Horstman D. H. Weiskopf R. Robinson S. (1979). The nature of the perception of effort at sea level and high altitude. Med. Sci. Sports 11, 150–154. 491872

[B75] Horvath S. M. Bedi J. F. Wagner J. A. Agnew J. (1988). Maximal aerobic capacity at several ambient concentrations of CO at several altitudes. J. Appl. Physiol. (1985) 65, 2696–2708. 10.1152/jappl.1988.65.6.2696 3215869

[B76] Howley E. T. Bassett D. R. Welch H. G. (1995). Criteria for maximal oxygen uptake: review and commentary. Med. Sci. Sports Exerc. 27, 1292–1301. 10.1249/00005768-199509000-00009 8531628

[B77] Hughson R. L. Green H. J. Sharratt M. T. (1995). Gas exchange, blood lactate, and plasma catecholamines during incremental exercise in hypoxia and normoxia. J. Appl. Physiol. (1985) 79, 1134–1141. 10.1152/jappl.1995.79.4.1134 8567554

[B78] Ibañez J. Rama R. Riera M. Prats M. T. Palacios L. (1993). Severe hypoxia decreases oxygen uptake relative to intensity during submaximal graded exercise. Eur. J. Appl. Physiol. 67, 7–13. 10.1007/BF00377696 8375369

[B79] Kacimi R. Richalet J. P. Corsin A. Abousahl I. Crozatier B. (1992). Hypoxia-induced downregulation of beta-adrenergic receptors in rat heart. J. Appl. Physiol. (1985) 73, 1377–1382. 10.1152/jappl.1992.73.4.1377 1447083

[B80] Kacimi R. Richalet J. P. Crozatier B. (1993). Hypoxia-induced differential modulation of adenosinergic and muscarinic receptors in rat heart. J. Appl. Physiol. (1985) 75, 1123–1128. 10.1152/jappl.1993.75.3.1123 8226520

[B81] Kaijser L. Grubbström J. Berglund B. (1990). Coronary circulation in acute hypoxia. Clin. Physiol. Oxf. Engl. 10, 259–263. 10.1111/j.1475-097X.1990.tb00094.x 2350942

[B82] Katayama K. Fujita O. Iemitsu M. Kawano H. Iwamoto E. Saito M.. (2013). The effect of acute exercise in hypoxia on flow-mediated vasodilation. Eur. J. Appl. Physiol. 113, 349–357. 10.1007/s00421-012-2442-5 22729610

[B83] Katayama K. Sato K. Hotta N. Ishida K. Iwasaki K. Miyamura M. (2007). Intermittent hypoxia does not increase exercise ventilation at simulated moderate altitude. Int. J. Sports Med. 28, 480–487. 10.1055/s-2006-955895 17357965

[B84] Kayser B. Narici M. Binzoni T. Grassi B. Cerretelli P. (1994). Fatigue and exhaustion in chronic hypobaric hypoxia: influence of exercising muscle mass. J. Appl. Physiol. (1985) 76, 634–640. 10.1152/jappl.1994.76.2.634 8175572

[B85] Keramidas M. E. Stavrou N. A. Kounalakis S. N. Eiken O. Mekjavic I. B. (2016). Severe hypoxia during incremental exercise to exhaustion provokes negative post-exercise affects. Physiol. Behav. 156, 171–176. 10.1016/j.physbeh.2016.01.021 26802281

[B86] Kjaer M. Bangsbo J. Lortie G. Galbo H. (1988). Hormonal response to exercise in humans: influence of hypoxia and physical training. Am. J. Physiol. 254, R197–R203. 10.1152/ajpregu.1988.254.2.R197 2830794

[B87] Kjaer M. Hanel B. Worm L. Perko G. Lewis S. F. Sahlin K.. (1999). Cardiovascular and neuroendocrine responses to exercise in hypoxia during impaired neural feedback from muscle. Am. J. Physiol. 277, R76–R85. 10.1152/ajpregu.1999.277.1.R76 10409260

[B88] Knuttgen H. G. Saltin B. (1973). Oxygen uptake, muscle high-energy phosphates, and lactate in exercise under acute hypoxic conditions in man. Acta Physiol. Scand. 87, 368–376. 10.1111/j.1748-1716.1973.tb05401.x 4697150

[B89] Koistinen P. Takala T. Martikkala V. Leppäluoto J. (1995). Aerobic fitness influences the response of maximal oxygen uptake and lactate threshold in acute hypobaric hypoxia. Int. J. Sports Med. 16, 78–81. 10.1055/s-2007-972968 7751080

[B90] Kong Z. Zang Y. Hu Y. (2014). Normobaric hypoxia training causes more weight loss than normoxia training after a 4-week residential camp for obese young adults. Sleep Breath. Schlaf Atm. 18, 591–597. 10.1007/s11325-013-0922-4 24318688

[B91] Lamberts R. P. Lemmink K. A. Durandt J. J. Lambert M. I. (2004). Variation in heart rate during submaximal exercise: implications for monitoring training. J. Strength Cond. Res. 18, 641–645. 10.1519/1533-4287(2004)18<641:VIHRDS>2.0.CO;2 15320683

[B92] Lawler J. Powers S. K. Thompson D. (1988). Linear relationship between VO2max and VO2max decrement during exposure to acute hypoxia. J. Appl. Physiol. (1985) 64, 1486–1492. 10.1152/jappl.1988.64.4.1486 3378983

[B93] Leone R. J. Lalande S. (2017). Intermittent hypoxia as a means to improve aerobic capacity in type 2 diabetes. Med. Hypotheses 100, 59–63. 10.1016/j.mehy.2017.01.010 28236850

[B94] Leuenberger U. Gleeson K. Wroblewski K. Prophet S. Zelis R. Zwillich C.. (1991). Norepinephrine clearance is increased during acute hypoxemia in humans. Am. J. Physiol. 261, H1659–H1664. 10.1152/ajpheart.1991.261.5.H1659 1951753

[B95] Levine B. D. (2015). Going high with heart disease: the effect of high altitude exposure in older individuals and patients with coronary artery disease. High Alt. Med. Biol. 16, 89–96. 10.1089/ham.2015.0043 26060882

[B96] Levine B. D. Stray-Gundersen J. (1997). “Living high-training low”: effect of moderate-altitude acclimatization with low-altitude training on performance. J. Appl. Physiol. (1985) 83, 102–112. 9216951 10.1152/jappl.1997.83.1.102

[B97] Levy M. N. (1971). Sympathetic-parasympathetic interactions in the heart. Circ. Res. 29, 437–445. 10.1161/01.RES.29.5.437 4330524

[B98] Lhuissier F. J. Canouï-Poitrine F. Richalet J. P. (2012). Ageing and cardiorespiratory response to hypoxia. J. Physiol. 590, 5461–5474. 10.1113/jphysiol.2012.238527 22907053 PMC3515831

[B99] Lühker O. Berger M. M. Pohlmann A. Hotz L. Gruhlke T. Hochreiter M. (2017). Changes in acid-base and ion balance during exercise in normoxia and normobaric hypoxia. Eur. J. Appl. Physiol. 117, 2251–2261. 10.1007/s00421-017-3712-z 28914359 PMC5640730

[B100] Lundby C. Araoz M. van Hall G. (2001a). Peak heart rate decreases with increasing severity of acute hypoxia. High Alt. Med. Biol. 2, 369–376. 10.1089/15270290152608543 11682016

[B101] Lundby C. Millet G. P. Calbet J. A. Bärtsch P. Subudhi A. W. (2012). Does “altitude training” increase exercise performance in elite athletes? Br. J. Sports Med. 46, 792–795. 10.1136/bjsports-2012-091231 22797528

[B102] Lundby C. Møller P. Kanstrup I. L. Olsen N. V. (2001b). Heart rate response to hypoxic exercise: role of dopamine D2-receptors and effect of oxygen supplementation. Clin. Sci. (Lond). 101, 377–383. 10.1042/cs1010377 11566075

[B103] Lundby C. Saltin B. van Hall G. (2000). The “lactate paradox”, evidence for a transient change in the course of acclimatization to severe hypoxia in lowlanders. Acta Physiol. Scand. 170, 265–269. 11450136 10.1046/j.1365-201x.2000.00785.x

[B104] Lundby C. van Hall G. (2001). Peak heart rates at extreme altitudes. High Alt. Med. Biol. 2, 41–45. 10.1089/152702901750067909 11252697

[B105] Lundby C. Van Hall G. (2002). Substrate utilization in sea level residents during exercise in acute hypoxia and after 4 weeks of acclimatization to 4100 m. Acta Physiol. Scand. 176, 195–201. 10.1046/j.1365-201X.2002.01030.x 12392499

[B106] Mao T. Y. Fu L. L. Wang J. S. (2011). Hypoxic exercise training causes erythrocyte senescence and rheological dysfunction by depressed Gardos channel activity. J. Appl. Physiol. (1985) 111, 382–391. 10.1152/japplphysiol.00096.2011 21551009

[B107] Marconi C. Marzorati M. Grassi B. Basnyat B. Colombini A. Kayser B.. (2004). Second generation Tibetan lowlanders acclimatize to high altitude more quickly than Caucasians. J. Physiol. 556, 661–671. 10.1113/jphysiol.2003.059188 14766936 PMC1664949

[B108] Martin D. O'Kroy J. (1993). Effects of acute hypoxia on the VO2 max of trained and untrained subjects. J. Sports Sci. 11, 37–42. 8450584 10.1080/02640419308729961

[B109] Masschelein E. Van Thienen R. Thomis M. Hespel P. (2015). High twin resemblance for sensitivity to hypoxia. Med. Sci. Sports Exerc. 47, 74–81. 10.1249/MSS.0000000000000386 24870565

[B110] Masuda K. Okazaki K. Kuno S. Asano K. Shimojo H. Katsuta S. (2001). Endurance training under 2500-m hypoxia does not increase myoglobin content in human skeletal muscle. Eur. J. Appl. Physiol. 85, 486–490. 10.1007/s004210100471 11606019

[B111] Meeuwsen T. Hendriksen I. J. Holewijn M. (2001). Training-induced increases in sea-level performance are enhanced by acute intermittent hypobaric hypoxia. Eur. J. Appl. Physiol. 84, 283–290. 10.1007/s004210000363 11374111

[B112] Mendelowitz D. (1999). Advances in parasympathetic control of heart rate and cardiac function. News Physiol. Sci. Int. J. Physiol. Prod. Jointly Int. Union Physiol. Sci. Am. Physiol. Soc. 14, 155–161. 10.1152/physiologyonline.1999.14.4.155 11390842

[B113] Millet G. P. Brocherie F. Girard O. Wehrlin J. P. Troesch S. Hauser A.. (2016a). Commentaries on Viewpoint: time for a new metric for hypoxic dose? J. Appl. Physiol. (1985) 121, 356–358. 27451276 10.1152/japplphysiol.00460.2016

[B114] Millet G. P. Debevec T. Brocherie F. Malatesta D. Girard O. (2016b). Therapeutic use of exercising in hypoxia: promises and limitations. Front. Physiol. 7:224. 10.3389/fphys.2016.00224 27375500 PMC4902009

[B115] Millet G. P. Faiss R. Pialoux V. (2012). Point: hypobaric hypoxia induces different physiological responses from normobaric hypoxia. J. Appl. Physiol. (1985) 112, 1783–1784. 10.1152/japplphysiol.00067.2012 22267386

[B116] Millet G. P. Roels B. Schmitt L. Woorons X. Richalet J. P. (2010). Combining hypoxic methods for peak performance. Sports Med 40, 1–25. 10.2165/11317920-000000000-00000 20020784

[B117] Mollard P. Woorons X. Letournel M. Cornolo J. Lamberto C. Beaudry M.. (2007a). Role of maximal heart rate and arterial O2 saturation on the decrement of VO2max in moderate acute hypoxia in trained and untrained men. Int. J. Sports Med. 28, 186–192. 10.1055/s-2006-924215 17024632

[B118] Mollard P. Woorons X. Letournel M. Lamberto C. Favret F. Pichon A.. (2007b). Determinant factors of the decrease in aerobic performance in moderate acute hypoxia in women endurance athletes. Respir. Physiol. Neurobiol. 159, 178–186. 10.1016/j.resp.2007.06.012 17766196

[B119] Mollard P. Woorons X. Letournel M. Lamberto C. Favret F. Pichon A.. (2007c). Determinants of maximal oxygen uptake in moderate acute hypoxia in endurance athletes. Eur. J. Appl. Physiol. 100, 663–673. 10.1007/s00421-007-0457-0 17534646

[B120] Mounier R. Brugniaux J. V. (2012). Counterpoint: Hypobaric hypoxia does not induce different responses from normobaric hypoxia. J. Appl. Physiol. (1985) 112, 1784–1786. 10.1152/japplphysiol.00067.2012a 22589489

[B121] Mourot L. Bouhaddi M. Gandelin E. Cappelle S. Nguyen N. U. Wolf J. P.. (2007). Conditions of autonomic reciprocal interplay versus autonomic co-activation: Effects on non-linear heart rate dynamics. Auton. Neurosci. 137, 27–36. 10.1016/j.autneu.2007.06.284 17662671

[B122] Mucci P. Blondel N. Fabre C. Nourry C. Berthoin S. (2004). Evidence of exercise-induced O2 arterial desaturation in non-elite sportsmen and sportswomen following high-intensity interval-training. Int. J. Sports Med. 25, 6–13. 10.1055/s-2003-45225 14750006

[B123] Naeije R. (2010). Physiological adaptation of the cardiovascular system to high altitude. Prog. Cardiovasc. Dis. 52, 456–466. 10.1016/j.pcad.2010.03.004 20417339

[B124] Netzer N. C. Chytra R. Küpper T. (2008). Low intense physical exercise in normobaric hypoxia leads to more weight loss in obese people than low intense physical exercise in normobaric sham hypoxia. Sleep Breath. Schlaf Atm. 12, 129–134. 10.1007/s11325-007-0149-3 18057976 PMC2276561

[B125] Nishiwaki M. Kawakami R. Saito K. Tamaki H. Takekura H. Ogita F. (2011). Vascular adaptations to hypobaric hypoxic training in postmenopausal women. J. Physiol. Sci. 61, 83–91. 10.1007/s12576-010-0126-7 21181322 PMC10717072

[B126] Noakes T. D. Peltonen J. E. Rusko H. K. (2001). Evidence that a central governor regulates exercise performance during acute hypoxia and hyperoxia. J. Exp. Biol. 204, 3225–3234. 11581338 10.1242/jeb.204.18.3225

[B127] Noordhof D. A. Schoots T. Hoekert D. H. de Koning J. J. (2013). Is gross efficiency lower at acute simulated altitude than at sea level? Int. J. Sports Physiol. Perform. 8, 319–322. 10.1123/ijspp.8.3.319 23070876

[B128] Ofner M. Wonisch M. Frei M. Tschakert G. Domej W. Kröpfl J. M.. (2014). Influence of acute normobaric hypoxia on physiological variables and lactate turn point determination in trained men. J. Sports Sci. Med. 13, 774–781. 25435769 PMC4234946

[B129] Ogawa T. Calbet J. A. Honda Y. Fujii N. Nishiyasu T. (2010). The effects of breathing a helium-oxygen gas mixture on maximal pulmonary ventilation and maximal oxygen consumption during exercise in acute moderate hypobaric hypoxia. Eur. J. Appl. Physiol. 110, 853–861. 10.1007/s00421-010-1570-z 20623231

[B130] Osawa T. Kime R. Hamaoka T. Katsumura T. Yamamoto M. (2011). Attenuation of muscle deoxygenation precedes EMG threshold in normoxia and hypoxia. Med. Sci. Sports Exerc. 43, 1406–1413. 10.1249/MSS.0b013e3182100261 21266933

[B131] Parati G. Agostoni P. Basnyat B. Bilo G. Brugger H. Coca A.. (2018). Clinical recommendations for high altitude exposure of individuals with pre-existing cardiovascular conditions. Eur. Heart J. 39, 1546–1554. 10.1093/eurheartj/ehx720 29340578 PMC5930248

[B132] Park H.-Y. Lim K. (2017). The effects of aerobic exercise at hypoxic condition during 6 weeks on body composition, blood pressure, arterial stiffness, and blood lipid level in obese women. Int. J. Sports Sci. Med. 1, 1–5.

[B133] Peltonen J. E. Rantamaki J. Niittymaki S. P. Sweins K. Viitasalo J. T. Rusko H. K. (1995). Effects of oxygen fraction in inspired air on rowing performance. Med. Sci. Sports Exerc. 27, 573–579. 10.1249/00005768-199504000-00016 7791589

[B134] Peltonen J. E. Rusko H. K. Rantamäki J. Sweins K. Niittymäki S. Viitasalo J. T. (1997). Effects of oxygen fraction in inspired air on force production and electromyogram activity during ergometer rowing. Eur. J. Appl. Physiol. 76, 495–503. 10.1007/s004210050281 9404860

[B135] Peltonen J. E. Tikkanen H. O. Rusko H. K. (2001). Cardiorespiratory responses to exercise in acute hypoxia, hyperoxia and normoxia. Eur. J. Appl. Physiol. 85, 82–88. 10.1007/s004210100411 11513325

[B136] Périard J. D. Racinais S. (2016). Performance and pacing during cycle exercise in hyperthermic and hypoxic conditions. Med. Sci. Sports Exerc. 48, 845–853. 10.1249/MSS.0000000000000839 26656777

[B137] Perri M. G. Anton S. D. Durning P. E. Ketterson T. U. Sydeman S. J. Berlant N. E.. (2002). Adherence to exercise prescriptions: effects of prescribing moderate versus higher levels of intensity and frequency. Health Psychol. Off. J. Div. Health Psychol. Am. Psychol. Assoc. 21, 452–458. 10.1037/0278-6133.21.5.452 12211512

[B138] Pescatello L. S. Franklin B. A. Fagard R. Farquhar W. B. Kelley G. A. Ray C. A.. (2004). American college of sports medicine position stand. exercise and hypertension. Med. Sci. Sports Exerc. 36, 533–553. 10.1249/01.MSS.0000115224.88514.3A 15076798

[B139] Pescatello L. S. MacDonald H. V. Lamberti L. Johnson B. T. (2015). Exercise for hypertension: a prescription update integrating existing recommendations with emerging research. Curr. Hypertens. Rep. 17:87. 10.1007/s11906-015-0600-y 26423529 PMC4589552

[B140] Pesta D. Hoppel F. Macek C. Messner H. Faulhaber M. Kobel C.. (2011). Similar qualitative and quantitative changes of mitochondrial respiration following strength and endurance training in normoxia and hypoxia in sedentary humans. Am. J. Physiol. Regul. Integr. Comp. Physiol. 301, R1078–R1087. 10.1152/ajpregu.00285.2011 21775647

[B141] Ponsot E. Dufour S. P. Doutreleau S. Lonsdorfer-Wolf E. Lampert E. Piquard F.. (2010). Impairment of maximal aerobic power with moderate hypoxia in endurance athletes: do skeletal muscle mitochondria play a role? Am. J. Physiol. Regul. Integr. Comp. Physiol. 298, R558–R566. 10.1152/ajpregu.00216.2009 20007521

[B142] Pramsohler S. Burtscher M. Faulhaber M. Gatterer H. Rausch L. Eliasson A.. (2017). Endurance training in normobaric hypoxia imposes less physical stress for geriatric rehabilitation. Front. Physiol. 8:514. 10.3389/fphys.2017.00514 28785224 PMC5517449

[B143] Puthon L. Bouzat P. Robach P. Favre-Juvin A. Doutreleau S. Verges S. (2017). Effect of ageing on hypoxic exercise cardiorespiratory, muscle and cerebral oxygenation responses in healthy humans. Exp. Physiol. 102, 436–447. 10.1113/EP085949 28130844

[B144] Richalet J. P. (1990). The heart and adrenergic system in hypoxia in Hypoxia: The Adaptations, eds Sutton J. R. Coates G. Remmers J. E. (Philadelphia, PA: Pmph Bc Decker), 231–245.

[B145] Richalet J. P. Kacimi R. Antezana A. M. (1992). The control of cardiac chronotropic function in hypobaric hypoxia. Int. J. Sports Med. 13(Suppl. 1), S22–S24. 10.1055/s-2007-1024582 1336483

[B146] Richalet J. P. Mehdioui H. Rathat C. Vignon P. Keromes A. Herry J. P.. (1988). Acute hypoxia decreases cardiac response to catecholamines in exercising humans. Int. J. Sports Med. 9, 157–162. 10.1055/s-2007-1024997 3384521

[B147] Richard N. A. Sahota I. S. Widmer N. Ferguson S. Sheel A. W. Koehle M. S. (2014). Acute mountain sickness, chemosensitivity, and cardiorespiratory responses in humans exposed to hypobaric and normobaric hypoxia. J. Appl. Physiol. (1985) 116, 945–952. 10.1152/japplphysiol.00319.2013 23823153

[B148] Roach R. C. Calbet J. A. Olsen N. V. Poulsen T. D. Vissing S. F. Saltin B. (1996). Peak exercise heart rate at high altitude. Med. Sci. Sport Exerc. 28.

[B149] Robergs R. A. Landwher R. (2002). The surprising history of the “HRmax = 220-age” equation. J. Exerc. Physiol. 5.

[B150] Robergs R. A. Quintana R. Parker D. L. Frankel C. C. (1998). Multiple variables explain the variability in the decrement in VO2max during acute hypobaric hypoxia. Med. Sci. Sports Exerc. 30, 869–879. 9624645 10.1097/00005768-199806000-00015

[B151] Robergs R. A. Quitana R. Parker D. Frankel C. C. (1997). Gender specific decrement in VO2max with increasing hypobaric hypoxia. Med. Sci. Sports Exerc. 135 10.1097/00005768-199705001-00776 9624645

[B152] Roche F. Reynaud C. Pichot V. Duverney D. Costes F. Garet M.. (2003). Effect of acute hypoxia on QT rate dependence and corrected QT interval in healthy subjects. Am. J. Cardiol. 91, 916–919. 10.1016/S0002-9149(03)00040-7 12667592

[B153] Rodway G. W. Lovelace A. J. Lanspa M. J. McIntosh S. E. Bell J. Briggs B.. (2016). Sildenafil and exercise capacity in the elderly at moderate altitude. Wilderness Environ. Med. 27, 307–315. 10.1016/j.wem.2016.01.006 27116921

[B154] Roels B. Bentley D. J. Coste O. Mercier J. Millet G. P. (2007). Effects of intermittent hypoxic training on cycling performance in well-trained athletes. Eur. J. Appl. Physiol. 101, 359–368. 10.1007/s00421-007-0506-8 17636319

[B155] Rostrup M. (1998). Catecholamines, hypoxia and high altitude. Acta Physiol. Scand. 162, 389–399. 10.1046/j.1365-201X.1998.00335.x 9578385

[B156] Saeed O. Bhatia V. Formica P. Browne A. Aldrich T. K. Shin J. J.. (2012). Improved exercise performance and skeletal muscle strength after simulated altitude exposure: a novel approach for patients with chronic heart failure. J. Card. Fail. 18, 387–391. 10.1016/j.cardfail.2012.02.003 22555269

[B157] Saltin B. Grover R. F. Blomqvist C. G. Hartley L. H. Johnson R. L. (1968). Maximal oxygen uptake and cardiac output after 2 weeks at 4300m. J. Appl. Physiol. 25, 400–409. 10.1152/jappl.1968.25.4.400

[B158] Saugy J. J. Rupp T. Faiss R. Lamon A. Bourdillon N. Millet G. P. (2016). Cycling time trial is more altered in hypobaric than normobaric hypoxia. Med. Sci. Sports Exerc. 48, 680–688. 10.1249/MSS.0000000000000810 26559447

[B159] Savourey G. Launay J. C. Besnard Y. Guinet A. Travers S. (2003). Normo- and hypobaric hypoxia: are there any physiological differences? Eur. J. Appl. Physiol. 89, 122–126. 10.1007/s00421-002-0789-8 12665974

[B160] Schmid J. P. Nobel D. Brugger N. Novak J. Palau P. Trepp A.. (2015). Short-term high altitude exposure at 3454 m is well tolerated in patients with stable heart failure. Eur. J. Heart Fail. 17, 182–186. 10.1002/ejhf.227 25597947

[B161] Schmid J. P. Noveanu M. Gaillet R. Hellige G. Wahl A. Saner H. (2006). Safety and exercise tolerance of acute high altitude exposure (3454 m) among patients with coronary artery disease. Heart Br. Card. Soc. 92, 921–925. 10.1136/hrt.2005.072520 16339809 PMC1860700

[B162] Schmidt W. Brabant G. Kröger C. Strauch S. Hilgendorf A. (1990). Atrial natriuretic peptide during and after maximal and submaximal exercise under normoxic and hypoxic conditions. Eur. J. Appl. Physiol. 61, 398–407. 10.1007/BF00236059 2150372

[B163] Schreuder T. H. Nyakayiru J. Houben J. Thijssen D. H. Hopman M. T. (2014). Impact of hypoxic versus normoxic training on physical fitness and vasculature in diabetes. High Alt. Med. Biol. 15, 349–355. 10.1089/ham.2013.1144 25251929

[B164] Seals D. R. Johnson D. G. Fregosi R. F. (1991). Hypoxia potentiates exercise-induced sympathetic neural activation in humans. J. Appl. Physiol. (1985) 71, 1032–1040. 10.1152/jappl.1991.71.3.1032 1757298

[B165] Semenza G. L. (2009). Regulation of oxygen homeostasis by hypoxia-inducible factor 1. Physiol. Bethesda 24, 97–106. 10.1152/physiol.00045.2008 19364912

[B166] Serebrovskaya T. V. Xi L. (2016). Intermittent hypoxia training as non-pharmacologic therapy for cardiovascular diseases: practical analysis on methods and equipment. Exp. Biol. Med. (Maywood) 241, 1708–1723. 10.1177/1535370216657614 27407098 PMC4999622

[B167] Sharma S. (1990). Clinical, biochemical, electrocardiographic and noninvasive hemodynamic assessment of cardiovascular status in natives at high to extreme altitudes (3000m-5500m) of the Himalayan region. Indian Heart J. 42, 375–379. 2086444

[B168] Shephard R. J. Bouhlel E. Vandewalle H. Monod H. (1988). Peak oxygen intake and hypoxia: influence of physical fitness. Int. J. Sports Med. 9, 279–283. 10.1055/s-2007-1025022 3182158

[B169] Shi B. Watanabe T. Shin S. Yabumoto T. Matsuoka T. (2013). Effect of normobaric hypoxia on cardiorespiratory and metabolic risk markers in healthy subjects Bateer Shi, Tsuneo Watanabe^*^, Sohee Shin, Tamotsu Yabumoto, Toshio Matsuoka. Adv. Biosci. Biotechnol. 4, 340–345. 10.4236/abb.2013.43044

[B170] Shi B. Watanabe T. Shin S. Yabumoto T. Takemura M. Matsuoka T. (2014). Effect of hypoxic training on inflammatory and metabolic risk factors: a crossover study in healthy subjects. Physiol. Rep. 2:e00198. 10.1002/phy2.198 24744877 PMC3967681

[B171] Siebenmann C. Lundby C. (2015). Regulation of cardiac output in hypoxia. Scand. J. Med. Sci. Sports 25(Suppl. 4), 53–59. 10.1111/sms.12619 26589118

[B172] Squires R. W. Buskirk E. R. (1982). Aerobic capacity during acute exposure to simulated altitude, 914 to 2286 meters. Med. Sci. Sports Exerc. 14, 36–40. 10.1249/00005768-198201000-00007 7070255

[B173] Staempfli R. Schmid J. P. Schenker S. Eser P. Trachsel L. D. Deluigi C.. (2016). Cardiopulmonary adaptation to short-term high altitude exposure in adult Fontan patients. Heart Br. Card. Soc. 102, 1296–1301. 10.1136/heartjnl-2016-309682 27217067

[B174] Stenberg J. Ekblom B. Messin R. (1966). Hemodynamic response to work at simulated altitude, 4,000 m. J. Appl. Physiol. 21, 1589–1594. 10.1152/jappl.1966.21.5.1589 5923231

[B175] Subudhi A. W. Dimmen A. C. Roach R. C. (2007). Effects of acute hypoxia on cerebral and muscle oxygenation during incremental exercise. J. Appl. Physiol. 103, 177–183. 10.1152/japplphysiol.01460.2006 17431082

[B176] Subudhi A. W. Lorenz M. C. Fulco C. S. Roach R. C. (2008). Cerebrovascular responses to incremental exercise during hypobaric hypoxia: effect of oxygenation on maximal performance. Am. J. Physiol. Heart Circ. Physiol. 294, H164–H171. 10.1152/ajpheart.01104.2007 18032522

[B177] Subudhi A. W. Olin J. T. Dimmen A. C. Polaner D. M. Kayser B. Roach R. C. (2011). Does cerebral oxygen delivery limit incremental exercise performance? J. Appl. Physiol. (1985) 111, 1727–1734. 10.1152/japplphysiol.00569.2011 21921244 PMC3233884

[B178] Sutton J. R. Reeves J. T. Wagner P. D. Groves B. M. Cymerman A. Malconian M. K.. (1988). Operation Everest II: oxygen transport during exercise at extreme simulated altitude. J. Appl. Physiol. (1985) 64, 1309–1321. 10.1152/jappl.1988.64.4.1309 3132445

[B179] Thiersch M. Swenson E. R. Haider T. Gassmann M. (2017). Reduced cancer mortality at high altitude: the role of glucose, lipids, iron and physical activity. Exp. Cell Res. 356, 209–216. 10.1016/j.yexcr.2017.03.048 28344053

[B180] Torres-Peralta R. Morales-Alamo D. González-Izal M. Losa-Reyna J. Pérez-Suárez I. Izquierdo M. (2015). Task failure during exercise to exhaustion in normoxia and hypoxia is due to reduced muscle activation caused by central mechanisms while muscle metaboreflex does not limit performance. Front. Physiol. 6:414 10.3389/fphys.2015.00414 26793117 PMC4707284

[B181] Tulumen E. Khalilayeva I. Aytemir K. Ergun Baris Kaya F. E. Sinan Deveci O. Aksoy H.. (2011). The reproducibility of heart rate recovery after treadmill exercise test. Ann. Noninvasive Electrocardiol. Off. J. Int. Soc. Holter Noninvasive Electrocardiol. Inc. 16, 365–372. 10.1111/j.1542-474X.2011.00464.x 22008492 PMC6932741

[B182] Urdampilleta A. González-Muniesa P. Portillo M. P. Martínez J. A. (2012). Usefulness of combining intermittent hypoxia and physical exercise in the treatment of obesity. J. Physiol. Biochem. 68, 289–304. 10.1007/s13105-011-0115-1 22045452

[B183] Van Thienen R. Hespel P. (2016). Enhanced muscular oxygen extraction in athletes exaggerates hypoxemia during exercise in hypoxia. J. Appl. Physiol. (1985) 120, 351–361. 10.1152/japplphysiol.00210.2015 26607244

[B184] Ventura N. Hoppeler H. Seiler R. Binggeli A. Mullis P. Vogt M. (2003). The response of trained athletes to six weeks of endurance training in hypoxia or normoxia. Int. J. Sports Med. 24, 166–172. 10.1055/s-2003-39086 12740733

[B185] Vogiatzis I. Louvaris Z. Habazettl H. Athanasopoulos D. Andrianopoulos V. Cherouveim E.. (2011). Frontal cerebral cortex blood flow, oxygen delivery and oxygenation during normoxic and hypoxic exercise in athletes. J. Physiol. 589, 4027–4039. 10.1113/jphysiol.2011.210880 21727220 PMC3180000

[B186] Vogt M. Hoppeler H. (2010). Is hypoxia training good for muscles and exercise performance? Prog. Cardiovasc. Dis. 52, 525–533. 10.1016/j.pcad.2010.02.013 20417346

[B187] Wagner P. D. (2000). Reduced maximal cardiac output at altitude–mechanisms and significance. Respir. Physiol. 120, 1–11. 10.1016/S0034-5687(99)00101-2 10786640

[B188] Wang H. Zhang T. Zhu W. Wu H. Yan S. (2014). Acute effects of continuous and interval low-intensity exercise on arterial stiffness in healthy young men. Eur. J. Appl. Physiol. 114, 1385–1392. 10.1007/s00421-014-2869-y 24643430

[B189] Wang J. S. Wu M. H. Mao T. Y. Fu T. Hsu C. C. (2010). Effects of normoxic and hypoxic exercise regimens on cardiac, muscular, and cerebral hemodynamics suppressed by severe hypoxia in humans. J. Appl. Physiol. (1985) 109, 219–229. 10.1152/japplphysiol.00138.2010 20431021

[B190] Wee J. Climstein M. (2015). Hypoxic training: clinical benefits on cardiometabolic risk factors. J. Sci. Med. Sport 18, 56–61. 10.1016/j.jsams.2013.10.247 24268571

[B191] Wehrlin J. P. Hallén J. (2006). Linear decrease in.VO2max and performance with increasing altitude in endurance athletes. Eur. J. Appl. Physiol. 96, 404–412. 10.1007/s00421-005-0081-9 16311764

[B192] Werle E. O. Strobel G. Weicker H. (1990). Decrease in rat cardiac beta 1- and beta 2-adrenoceptors by training and endurance exercise. Life Sci. 46, 9–17. 10.1016/0024-3205(90)90051-R 2153886

[B193] West J. B. Boyer S. J. Graber D. J. Hackett P. H. Maret K. H. Milledge J. S.. (1983). Maximal exercise at extreme altitudes on Mount Everest. J. Appl. Physiol. 55, 688–698. 10.1152/jappl.1983.55.3.688 6415008

[B194] Wiesner S. Haufe S. Engeli S. Mutschler H. Haas U. Luft F. C.. (2010). Influences of normobaric hypoxia training on physical fitness and metabolic risk markers in overweight to obese subjects. Obesity (Silver Spring). 18, 116–120. 10.1038/oby.2009.193 19543214

[B195] Wilson E. N. Anderson M. Snyder B. Duong P. Trieu J. Schreihofer D. A.. (2018). Chronic intermittent hypoxia induces hormonal and male sexual behavioral changes: Hypoxia as an advancer of aging. Physiol. Behav. 189, 64–73. 10.1016/j.physbeh.2018.03.007 29526572 PMC5882542

[B196] Windsor J. S. Rodway G. W. Montgomery H. E. (2010). A review of electrocardiography in the high altitude environment. High Alt. Med. Biol. 11, 51–60. 10.1089/ham.2009.1065 20367489

[B197] Woods D. R. O'Hara J. P. Boos C. J. Hodkinson P. D. Tsakirides C. Hill N. E.. (2017). Markers of physiological stress during exercise under conditions of normoxia, normobaric hypoxia, hypobaric hypoxia, and genuine high altitude. Eur. J. Appl. Physiol. 117, 893–900. 10.1007/s00421-017-3573-5 28299447 PMC5388721

[B198] Woolcott O. O. Castillo O. A. Gutierrez C. Elashoff R. M. Stefanovski D. Bergman R. N. (2014). Inverse association between diabetes and altitude: a cross-sectional study in the adult population of the United States. Obesity (Silver Spring). 22, 2080–2090. 10.1002/oby.20800 24890677 PMC4149588

[B199] Woolcott O. O. Gutierrez C. Castillo O. A. Elashoff R. M. Stefanovski D. Bergman R. N. (2016). Inverse association between altitude and obesity: a prevalence study among andean and low-altitude adult individuals of Peru. Obesity (Silver Spring). 24, 929–937. 10.1002/oby.21401 26935008 PMC4814295

[B200] Woorons X. Mollard P. Lamberto C. Letournel M. Richalet J. P. (2005). Effect of acute hypoxia on maximal exercise in trained and sedentary women. Med. Sci. Sports Exerc. 37, 147–154. 10.1249/01.MSS.0000150020.25153.34 15632681

[B201] Young A. J. Evans W. J. Cymerman A. Pandolf K. B. Knapik J. J. Maher J. T. (1982). Sparing effect of chronic high-altitude exposure on muscle glycogen utilization. J. Appl. Physiol. 52, 857–862. 10.1152/jappl.1982.52.4.857 7085419

[B202] Zavorsky G. S. (2000). Evidence and possible mechanisms of altered maximum heart rate with endurance training and tapering. Sports Med. 29, 13–26. 10.2165/00007256-200029010-00002 10688280

