# Supplementary material for: Limitation of Maximal Heart Rate in Hypoxia: Mechanisms and Clinical Importance
Source: Front Physiol. 2018 Jul 23;9:972. doi: 10.3389/fphys.2018.00972 (PMC6064954; doi:10.3389/fphys.2018.00972)
Supplement: Supplementary file 1 [file Table_1.docx]

**Table S1**. Maximal heart rate response to exercise in acute (hypobaric or normorbaric) hypoxia.

| **Study** | **Participants** | **Maximal O_2_ uptake** | **Altitude** | **Incremental Exercise protocol** | **Main findings** |
| --- | --- | --- | --- | --- | --- |
| **Healthy subjects** | | | | | |
| (Agostoni et al., 2000) | Fourteen healthy males 57±13 yrs  Thirty-eight patients with clinically stable heart failure 61±7 yrs | Controls: 35.5±9.8 ml/kg/min  Patients: 22.1±2.3 ml/kg/min | Normobaric hypoxia  92, 1,000, 1,500, 2,000, and 3,000 m | Cycle ergometer | Controls:  92 m: 163 bpm  1,000 m: 146 bpm  1,500 m: 152 bpm  2,000 m: 143 bpm  3,000 m: 149 bpm  Patients:  92 m: 138 bpm  1,000 m: 120 bpm  1,500 m: 127 bpm  2,000 m: 125 bpm  3,000 m: 130 bpm  Recalculated from pulse O_2_ |
| (Angermann et al., 2006) | Seven male Nordic combined skiers  70.1±6.5 kg; 178±6.0 cm; 21.4±2.5 yrs | 57.3±3.7 ml/min/kg | Normobaric hypoxia  560 vs 3,200 m | Cycle ergometer (leg)  100 W (N) or 40 W (H) then 30 W every 2 min | 193±7 vs 188±8 bpm  Significantly reduced, as referred to table but no significant change specified in manuscript. |
| (Angermann et al., 2006) | Seven male Nordic combined skiers  70.1±6.5 kg; 178±6.0 cm; 21.4±2.5 yrs | 53.6±4.2 ml/min/kg | Normobaric hypoxia  560 vs 3,200 m | Double poling ergometer for cross-country Skiing  60 W (N) or 40 W (H) then 20 W every 2 min to loads just above anaerobic lactate threshold then 2.5-min then final bout to exhaustion at 60 W below then 20 W every 30 s | 192±10 vs 190±10 bpm |
| (Bailey et al., 2001) | Thirty-two males  22±3 yrs | 50±9 ml/kg/min | Normobaric hypoxia  0 vs 2,100m | Cycle ergometer  60 W then 30 W / 2 min | 189±7 vs 186±13 bpm |
| (Benoit et al., 1997) | Twenty-two males  65.8±6.5 kg; 176.6±6.5 cm; 22.8+6.5 yrs | 3.78±0.74 l/min | Normobaric hypoxia  0 vs 5,500m | Cycle ergometer  Normoxia: 80 or 100 W then 20 or 30 W / 2 min  Hypoxia: 50 or 60 W then 15 W / 2 min | 196±7 vs 180±8 bpm* |
| (Benoit et al., 2003) | Forty-six healthy males in three groups  High: 68.2±6.5 kg; 175.7±6.0 cm; 22.5±4.5 yrs  Medium: 69.5±8.7 kg; 175.7±5.6 cm; 22.4±1.7 yrs  Low: 68.9±12.2 kg; 172.6±9.8 cm; 22.6±2.4 yrs | High: 4.42±0.5 l/min  Medium: 3.47±0.62 l/min  Low: 2.88±0.70 l/min | Normobaric hypoxia  0 vs 5,500m | Cycle ergometer  Normoxia: 80 or 100 W then 20 or 30 W every 2 min  Hypoxia: 50 or 60 W then 15 W every 2 min | Low: 196.1±6.7 vs 184.3±7.2 bpm*  Medium: 194.4±6.9 vs 179.8±7.3 bpm*  High: 196.2±7.9 vs 178.7±6.0 bpm* |
| (Billat et al., 2003) | Eight males  65±5 kg; 178±4 cm;27±7 yrs | 57.3±3.3 ml/kg/min | Normobaric Hypoxia  0 vs 2,400 m | Treadmill  3 min at 12 km/h (10 in hypoxia) then 1 km/h / 1 min | 193±8 vs. 187±8 bpm * |
| (Bouissou et al., 1986) | Eight males  74±8 kg; 178±6 cm; 24±1 years | 55.5±1.3 ml/kg/min | Normobaric hypoxia  0 vs 3,000 m | Cycle ergometer | 185±6 vs 183±2 bpm |
| (Brothers et al., 2007) | Twenty-four healthy males (N = 13) and femalse (N = 11) trained subjects residing at 1500-2000 m  M: 72.1±4.3 kg; 181.6±5.8 cm; 27.7±4.5 yrs  F: 61.2±5.0 kg; 166.5±7.4m; 29.5±4.2 yrs | Males: 4.62±0.30 l/min  Females: 3.10±0.15 l/min | Hypobaric hypoxia  1,625 vs 2,100m | Cycle ergometer  150 (M) or 120 (F) W then 30 (M) or 20 (F) W / 3 min | Males: 184.8±9.1 vs 184.5±9.4 bpm  Females: 182.9±2.8 vs 183.5±5.2 bpm |
| (Calbet et al., 2003a) | Nine healthy males  74±4 kg; 176±3 cm; 24.3±0.5 yrs | 59±2 ml/kg/min for the males (n=5) and 53±4 ml/kg/min for the females (n=4) | Normobaric hypoxia  0 vs 5,500m | cycle ergometer  120 W then 40 W every 1 min | 182 vs 168 bpm* |
| (Calbet et al., 2003b) | Seven healthy males  74±4 kg; 176±3 cm; 24.0±0.6 yrs. | 61±0.5 for the males (n=3) and 50±3 ml/kg/min for the females (n=4) | Normobaric hypoxia  0 vs 5,500m | Cycle ergometer  120 W then 40 W every 1 min | 181 vs 169 bpm * |
| (Chapman et al., 1999) | Fourteen endurance trained males  Eight EIH: 67.7±2.5 kg; 181.4±3.9 cm; 21.4±1.4 yrs  Six Non EIH: 66.4±10.4 kg, 178.8±4.4 cm; 22.8±3.9 yrs | EIH: 71.1±5.3 ml/kg/min  Non-EIH: 67.2±7.6 ml/kg/min | Normobaric hypoxia  0 vs 1,000m | Treadmill  2 min at speed selected between 11.2 and 14.5 km/h at 0% then 2% increase every min | EIH:  0 m: 188.4±9.3 bpm  1,000 m: 187.9±8.2 bpm  Non-EIH:  0 m: 184.2±9.2 bpm  1,000 m: 184.7±9.3 bpm |
| (Davison et al., 2006) | Thirty healthy males  74±10 kg; 176±5.0 cm; 21.1±1.1 yrs | 47.8±7 ml/min/kg | Normobaric hypoxia  0 vs 2,200m | Cycle ergometer  0.5 kg at 80 rpm then 0.4 kg 2 mins (power output increase of 32 W/stage) | 195 ± 9 vs 193 ± 8 bpm |
| (Debevec et al., 2010) | Eighteen healthy, young moderately active  Nine H: 77.4±8.7 kg; 182.8±4.3 cm; ; 20.1±3.0 yrs  Nine N: 72.9±9.7 kg; 179.3±5.0 cm; 22.1±4.0 yrs | H: 48 ml/kg/min  N: 46 ml/kg/min  Estimated from graph | Normobaric Hypoxia  0 vs 4,500m | Cycle ergometer  60 W then 30 W every 2 min | Hpre: 189 vs 180 bpm  Hpost: 178 vs 172 bpm  Hpost 10 days: 178 vs 176 bpm  Npre: 191 vs 182 bpm  Npost: 186 vs 175 bpm  Npost 10 days: 187 vs 175 bpm |
| (Dekerle et al., 2012) | Five active males en and six females  73 ± 9 kg; 35±10 yrs | 3.5±0.4 l/min | Normobaric hypoxia  0 vs 2,500m | Cycle ergometer  50 W for 5 min and then 5 W every 12 s (equating to 25 W/min) | 187 ± 11 vs 179 ± 11 bpm* |
| (Dill et al., 1966) | Four males  81±8.5 kg; 182±5 cm; 39±24 yrs | 3.09 l/min | Hypobaric Hypoxia  0, 2,700, 3,800, 4,400 m | Cycle ergometer  150 kpm/min | 0 m: 182 bpm  2,700 m: 179 bpm  3,800 m: 177 bpm  4,400 m: 174 bpm* |
| (Drinkwater et al., 1979) | Eight females  63.6±2.6 kg, 168.6±2.1 cm, 35.6±4.0 yrs | 43.7±1.4 ml/kg/min | Normobaric Hypoxia  0 vs 4,100m | Treadmill  90m/min at 0% of slope then 1% / min | 184.7±4.7 vs 176.7±4.2 bpm* |
| (Dufour et al., 2006) | Eighteen male distance runners. Two groups trained in  Nine H : 70.6±2.2 and 71.3±2.2 kg; 180±1 yrs  Nine N: 180±2 cm; 30.3±6.3 and 30.3±6.1 yrs. | H: 64.2±1.2  N: 61.5±1.1 ml/min/kg | Normobaric hypoxia  300 vs 3,000 m | Treadmill  10 km/h then 1 km/h every 2-min | Pre vs post training  H in N: 183±2 vs 182±4 bpm  H in H: 170±3 vs 172±3 bpm  N in N: 184±4 vs 185±3 bpm  N in H: 174±4 vs 174±3 bpm  Hypoxic effect not tested |
| (Ekblom et al., 1975) | Nine healthy males  27 (23-34) yrs | 58 (52.5-54.3) ml/kg/min | Hypoxia  (CO rebreathing induced) | Treadmill (n=4)  Cycle ergometer (n=5) | 184±3.5 vs 182±3.1 bpm |
| (Escourrou et al. 1984) | Seven healthy males  65-82 kg; 22-32 yrs | 4.14 l/min | Normobaric hypoxia  0 vs 4,500 m | cycle ergometer | 185 vs 180 bpm* |
| (Fagraeus et al., 1973) | Twelve males  71.8±8.5 kg, 181.1±7.3 cm, 29.3±3.7 yrs | 3.7±0.7 l/min | Hypobaric Hypoxia  0 vs 3,000m | Cycle ergometer | 185.7 4.2 vs 184.0 3.1 bpm |
| (Faiss et al., 2014) | Twenty three Ski mountaineers (5 males, 6 females)  Twelve U: 69.4±8.0 kg; 175±5 cm;27±4 yrs  Eleven T: 63.1±5 kg; 173±3 cm; 28±8yrs | U: 47.3 ± 4.3 ml/kg/min  T: 63.0 ± 9.0 ml/kg/min | Normobaric hypoxia  485 vs. 3,000m | Treadmill  1.5 m.s^-1^ then 0.5 m/s every 3 min until 4 m.s^-1^ with the slope of the treadmill set at 2%. When 4 m/s reached, slope was increased by 2% every 3 min | U: 193 ± 11 vs 187 ± 11 bpm  T: 185 ± 13 180 ± 12 bpm |
| (Ferretti et al., 1997) | Ten healthy males divided in  Five U: 73.6+6.1kg; 1.77+0.06 m;33 + 9 yrs  Five T: 66.3+4.4 kg; 1.76+0.04 m; 25 + 7 yrs | U: 42.1±2.6 ml/kg/min  T: 62.1±1.8 ml/kg/min | Normobaric hypoxia  0, 1,200, 2,200, 3,700, 5,000m | cycle ergometer  50 and 100 W for U and T then 50W/5 min then 25W/5min | U:  0 m: 190±5 bpm  1,200: m 186±7 bpm  2,200 m: 183±9 bpm  3,700 m: 179±8 bpm  5,000 m: 167±10 bpm  T:  0 m: 192±4 bpm  1,200 m: 187±2 bpm  2,200 m: 188±4 bpm  3,700 m: 186±4 bpm  5,000 m: 180±3 bpm |
| (Friedmann et al., 2004) | Eleven trained males  73.2±6.8 kg; 183.4±5.0 cm26.1±5.3 yrs | 67.8±6.4 ml/kg/min | Normobaric hypoxia 0 vs 2,500 m | Treadmill 2% inclination  6 km/h then 2km/h every 3 min | 193±7 vs 186±8 bpm* |
| (Fukuda et al., 2010) | Nine healthy males  66.0±1.7 kg; 170.8±2.1 cm; 26.9±1.5 yrs | 46.4±2.5 ml/min/kg | Normobaric hypoxia  0 vs 3,000 m | Cycle ergometer  50 W for 4 min then 1 W every 3 sec | 182±3 vs 181±2 bpm |
| (Gallagher et al., 2015) | Fifteen healthy subjects (seven women and eight men)  72.8±14.0 kg; 176.4±10.2 cm; 22±2 years | 46.3±7.0 ml/kg/min | Normobaric hypoxia  1,000, 2,000, 3,000 and 4,000 m | Cycle ergometer  50 W then 20 W for women and 25 W for men every 1 min | SL: 182±13  1,000 m: 178±11 bpm  2,000 m: 177±9 bpm  3,000 m: 178±9 bpm  4,000 m: 176±11 pm |
| (Gore et al., 1996) | Eleven Trained males  71.4±1.1 kg; 179.4±1.5 cm; 23.3±1.5 yrs  Nine Untrained males  78.1±2.2 kg; 179.5±1.6 cm; 27.1±2.8 yrs | U: 51±3 ml/kg/min  T: 77±1 ml/kg/min | Hypobaric hypoxia  168 vs 748 m | Cycle ergometer  Trained: 200W then 25W / min  Untrained: 100W then 25W /min | T: 190±3 vs 186±3 bpm  U: 189±3 vs 189±3 bpm |
| (Grataloup et al., 2007) | Nineteen healthy trained males  EIH: 68.3± 3.7 kg; 180.5±6.1kg; 19.6±1.1 yrs  Non-EIH: 67.2±8.5 kg; 178.9±5.1 cm; 23.3±5.3 yrs | Non-EIH: 62.8±2.6 ml/kg/min  EIH: 63.2±3.1 ml/kg/min | Normobaric hypoxia  0 vs 2,300m | Cycle ergometer  2 min unload then 23±2W every 1 min· | Non-EIH:  N vs H : 191±8 vs 186±8 bpm*  EIH:  N vs H : 189±8 vs 181±7 bpm* |
| (Haufe et al., 2008) | Twenty healthy males  H: 82.5±12.0 kg; 1.8±0.1m; 29±5.9 yrs  N:78±7.5 kg; 1.8±0.1m; 28.1±5.2 yrs | H: 47±3.4 ml/min/kg  N: 44±6.2 ml/min/kg | Normobaric hypoxia  0 vs 2,740m | Treadmill  2.5 km/h than 0.3 km/h / 5 min at 0% | Npre: 195±6.3 vs 192±3.6 bpm  Npost: 191±8.9 vs 188±7.5 bpm  Hpre: 195±10 vs 189±9.9 bpm  Hpost: 189±11 vs 185±11 bpm |
| (Heubert et al., 2005) | Nine trained males  69.8±1.9 kg; 179.4±2.2 cm; 27±3 yrs | 62.7±2 ml/kg/min | Normobaric hypoxia  0 vs 2,500m | Cycle ergometer  80W then 40W every 3 min | 187±1 vs 183±2 bpm |
| (Horiuchi et al., 2017) | Nine healthy males  73±9 kg; 176±5 cm, 21±3 yrs | 3.127±3.61 l/min | Normobaric hypoxia  0 vs 3,200m | Cycle ergometer  30 W then 30 W every 1 min | 180±9 vs 178±6 bpm |
| (Hogan et al., 1983) | Six males  73.1±4.5 kg; 27±3 yrs | 46.8±3.0 ml/kg/min | Normobaric hypoxia  0 vs 1,800 m | Cycle ergometer  105 W and increasing by 25 W every 3 min | 183±3 vs 184±3bpm |
| (Horvath et al., 1988) | Eight males  75.6±12.2 kg; 182±1cm; 23.2±4.9 yrs  Eight females  56.3±6.2 kg; 165±1 cm; 24.8±5.9 yrs | Males: 51.4±5.1 ml/kg/min  Females: 39.9±5.4 ml/kg/min | Hypobaric hypoxia (climatic chamber)  55, 1,524, 2,134, 3,048m | Cycle ergometer  50 W then 25 W every min | Males  55 m: 184.2±16.5 bpm  1,524 m: 186.4±19.9 bpm  2,134 m:183.8±16.6 bpm  3,048 m:180.4±16.2 bpm  Females  55 m: 185.8±6.4 bpm  1,524 m: 185.8±11.3 bpm  2,134 m: 182.5±7.6 bpm  3,048 m:185.3±9.6 bpm |
| (Hughson et al., 1995) | Nine healthy males | 4164±184 ml/min | Normobaric hypoxia  0 vs 3,000 m | Cycle ergometer  25 W then 15 W every min | 195±2 vs. 191±6 bpm |
| (Ibañez et al., 1993) | Nine healthy males  66.9±8.3 kg; 1,76±0.07 m; 27±2.9 yrs | 34.1±2.8 ml/kg/min | Normobaric hypoxia  0 vs 5,500m | Cycle ergometer  75 W then 25 W / 2 min | 185±7 vs 172±8 bpm* |
| (Katayama et al., 2007) | Eighteen trained males divided in three groupes  G1: 57.6 ± 2.9 kg; 171.0 ± 6.4 cm; 20.3 ± 0.8 yrs  G2: 57.7 ± 2.6 kg; 170.5 ± 2.4 cm; 19.8 ± 1.8 yrs  G3: 60.3 ± 5.3 kg; 170.9 ± 3.8 cm; 21.0 ± 1.1 yrs | Group 1: 63.9 ± 2.3  Group 2: 63.1 ± 2.6  Group 3: 65.9 ± 2.7  ml/kg/min | Normobaric hypoxia  0 vs 2,500m | 120W (N) or 90 (H) then 30W / 2 min | G1: 185.3 ± 8.1 vs 179.2 ± 15.2 bpm  G2: 183.8 ± 16.4 vs 180.5 ± 8.8 bpm  G3: 189.0 ± 4.4 vs 185.0 ± 5.9 bpm |
| (Katayama et al., 2013) | Eight healthy males  23.8±2.0 years, 177.3±1.6 cm, and 71.5±1.9 kg. | 31.3±1.3 ml/kg/min | Normobaric Hypoxia  0 vs 4,500m | arm cycling ergometer  10 W than 5 W every min | 177.0 ± 4.3 vs 174.9 ± 3.5 bpm |
| (Keramidas et al., 2016) | Thirty-six healthy males  74.0±6.3 kg; 179.9±5.7 cm; 22.6±3.5 yrs | 52.4±8.9 ml/kg/min | Normobaric hypoxia  0 vs 4,500 m | Cycle ergometer  60 W then 30 W every 1 min | 187±7 vs 180±7 bpm * |
| (Kjaer et al., 1988) | Seven untrained males  71 (58-80) kg; 178 (165-187) cm; 25(22-27) yrs  Seven endurance-trained males  71 (60-80)kg; 180 (165-186) cm; 25(23-30) yrs | U: 48 (45-52) ml/kg/min  T: 66 (61-70) ml/kg/min | Hypobaric hypoxia  0 vs 4,200 m | Cycle ergometer | U: 192±3 vs 190±3 bpm*  T: 185±1 vs 180±2 bpm* |
| (Koistinen et al., 1995) | Twelve trained males athletes  73.4±1 1.4kg; 178.4±10.5 cm; 20.7±2.3 yrs | 57.4±7.1 ml/kg/min | Hypobaric Hypoxia  0 vs 3,000m | Cycle ergometer  50W every 4 min | 185±5.9 vs 179±4.6 bpm* |
| (Knuttgen and Saltin, 1973) | Five males  79.6±3.5 kg, 185.4±6.0 cm, 23.2±2.2 yrs | 4.1±0.4 l/min | Hypobaric Hypoxia  0 vs 4,000m | Cycle ergometer | 170 vs 172 bpm |
| (Lawler et al., 1988) | Thirteen males  74±9.5 kg; 177±4.4 cm; 25.7±4.1 yrs | 6 U: 45.0±2.2  7 T: 64.5±2.4 ml/kg/min | Normobaric hypoxia  0 vs 3000m | Cycle ergometer  35 W then 35 W every min | Trained: 182±3 vs 180±2 bpm  Untrained:187±5 vs184±5 bpm |
| (Lundby et al., 2001a) | Five males  77.6 (73 to 83) kg; 1.81 (1.75 to 1.87) m; 24.0 (21 to 27) yrs | NA | Hypobaric hypoxia  0, 3,300, 4,300, 5,300, and 6,300 m of altitude | Cycle ergometer  Normoxia: 40 W the 80 W every 2’30”  Hypoxia: Unload then 80 W every 2’30” | SL: 191.6±3.3  3,300 m: 189.6±3.4 bpm*  4,300 m: 182.6±3.0 bpm*  5,300 m: 175.6±2.9 bpm*  6,300m: 165.6±1.3 bpm* |
| (Lundby et al., 2001b) | 12 subjects (five females and seven males)  73.5±0.8 kg; 179.2±4.3 cm; 26.1±1.4 yrs | 3.9±0.2 l/min | Hypobaric hypoxia  0 vs 4,559 m | Cycle ergometer  40 W every 1 min | SL: 188±3  **After 1 day: 175±5*** |
| (Lundby and van Hall, 2001) | Five males  84.6 (75.0–91.5) kg; 1.84 (1.77–1.89) m; 32.6 (25–43) yrs | 53.9 (47.6–61.3) ml/kg/min | Normobaric hypoxia  0 vs 5,400 m | Cycle ergometer  Unloaded then 40 W every 1.5 | SL: 186±5 bpm  5,400 m: 170±12 bpm* |
| (Lundby and Van Hall, 2002) | Eight trained males  186 ± 2 cm; 78±3 kg; 25 ± 1 yrs | 4.2±0.3 l/min | Hypobaric Hypoxia  0 vs 4,100 m | Cycle ergometer  100 W then 40 W / 2,5 min | 187 ± 8 vs 176 ± 8 bpm* |
| (Lühker et al., 2017) | Ninetheen males  74.5 ± 2 kg; 178 ± 2 cm; 36 ± 3 yrs | NA | Normobaric hypoxia  0 vs 4,500m | Cycle ergometer  50 W then 50 W / 3 min | 192±3 vs 180±3 bpm* |
| (Marconi et al., 2004) | Eight Tibetans  57.3±8.5 kg; 170±8 cm; 20±2 yrs  Ten Untrained Caucasians  82.8±12.5 kg, 179±9 cm; 34±7 yrs  Five Trained Caucasians  65.9±5.6 kg; 173±1 cm; 32±4 yrs | Tibetans: 4.3±0.7 l/min  Caucasians U: 3.7±0.4 l/min  Caucasians T: 4.3±0.4 l/min | Hypobaric hypoxia  Untrained and trained Caucasians 122 m vs 5,050 m  Tibetans: 1300 m vs 5,050 m | 30-90 W (depending on altitude, age, body mass and Fitness) then 30W every 3 min | Tibetans: 188±13 vs 175±8 bpm *  Caucasians U: 178±13 vs 152±15 bpm *  Caucasians T: 190±7 vs 161±10 bpm* |
| (Martin and O’Kroy, 1993) | Eight Trained males  67.4 ± 6.1; 178.1±7.1; 25.0 ± 3.8  Eight Untrained males  76.7±16.2; 181.1 ±8.9; 22.6 ± 3.0 | U: 45.4±5.5 ml/kg/min T: 67.2±4.0 ml/kg/min | Normobaric Hypoxia  0 vs 3,600 m | Cycle ergometer  70 W then 35 W / 2 min | U: 189±6 vs 189±4 bpm  T: 191±5 vs 183±7 bpm * |
| (Masschelein et al., 2015) | Fifteen males  72.3 ±2.5 kg; 21.1±1.0 yrs | 61.7±2.1 ml/kg/min | Normobaric hypoxia  0 vs 5,000m | Cycle ergometer  50 W then 20 W/min | 191±3 vs 173±2 bpm* |
| (Masschelein et al., 2015) | Thirteen Caucasian monozygotic twin brothers  23.8±1.8 kg/m²; 24.9±4.4 yrs | 54.7±8.1 ml/kg/min | Normobaric hypoxia  0 vs 5,300m | Cycle ergometer  50 W than 20 W every min | 190±9 vs 175±10 bpm* |
| (Mollard et al., 2007c) | Sixteen males triathletes  Untrained  69.9±2.5 kg; 1.75±0.20 m; 25.0±1.5 yrs  Trained 71.5±2.9 kg; 1.78±0.31 m; 28.6±1.0 yrs | U: 43.3±2.1 ml/kg/min  T: 65.5±1.8 ml/kg/min. | Normobaric hypoxic  0, 1,000, 2,500, 4,500m | cycle ergometer  60 W then 30 W every 2 min | U  SL:192  1,000 m: 186 bpm*  2,500 m: 185 bpm*  4,500 m: 184 bpm*  T  SL: 180  1,000 m: 177 bpm  2,500 m: 176 bpm*  4,500 m: 168 bpm* |
| (Mollard et al., 2007b) | Sixteen women  Untrained  53.3±5.0 kg; 1.64±4.8 m; 27.1±3.8 yrs  Trained  57.9±6.6 kg; 1.68±4.0 m; 28.4±4.7 yrs | U: 37.7±1.9 ml/kg/min  T: 55.0±1.2 ml/kg/min. | Normobaric hypoxic  0, 1,000, 2,500, 4,500m | Cycle ergometer  40 W then 25 W every 2 min | U  SL: 183.2±3.2 bpm  1,000 m: 182.1±4.2 bpm  2,500 m:182.1±3.1 bpm  4,500 m:177.1±3.2 bpm  T  SL: 178.7±4.6 bpm  1,000 m: 178.1±5.2 bpm  2,500 m:175.2±4.6 bpm  4500 m:168.7±3.8* |
| (Mollard et al., 2007a) | Seventeen males  Untrained  73.2±7.5 kg; 1.78±7.5 m; 27.6±6.5 yrs  Trained  78.1±7.9 kg; 1.78±2.9 m; 27.8±3.3 yrs | Untrained: 44.1±5.3 ml/kg/min;  Trained: 65.5± 3.1 ml/kg/min | Normobaric hypoxia  0, 1,000, 1,500, 2,500, 3,500, 4,500m | Cycle ergometer  60 W followed by an incremental load of 30 W every 2 min | U  SL:191± 7.1  1,000 m: 187.7± 6.4  1,500 m: 189.6±9.9  2,500 m:186.8±12 bpm*  3,500 m: 186± 8 bpm*  4,500 m: 188±9.4 bpm*  T  SL: 185±8 bpm  1,000 m:181±6.9 bpm*  1,500 m: 178±7.7 bpm*  2,500 m: 178±6.3 bpm*  3,500 m: 174±6.6 bpm*  4,500 m: 170±3.8 bpm* |
| (Peltonen et al., 2001) | Six trained males  73.6±5.0 kg; 181.1±3.4 cm; 24±5 yrs | 4.55±0.32 L/min | Normobaric hypoxia  0 vs 2,500 m | Cycle ergometer  100 W every 5 min until 400 W and 50 W every 5min thereafter | 181±11 vs 177±15 bpm* |
| (Périard and Racinais, 2016) | Twelve trained males: 77.3±5.1 kg; 177.3±6.1 cm; 35±4 yrs | 4.6±0.3 L/min | Norm,obaric hypoxia  0 vs 3000 m | Cycle ergometer  100 W and increasing by 25 W/min | 182±8 bpm vs 178±8 bpm* |
| (Puthon et al., 2017) | Sixteen young  68±9 kg; 172±10cm; 25±2 yrs  Fifteen older males  66±10 kg; 167±10 cm; 65±3 yrs | Young: 43.7±7.0 ml/min/kg  Old: 34.1±7.2 ml/min/kg | Normobaric hypoxia  0 vs 4,500m | Cycle ergometer  N: 60 or 80 W then 10, 15 or 20 W / 2 min  H: 40 or 60 W then 10 or 15 W / 2 min | Young: 181±10 vs 165±10 bpm  Old: 159±11 vs 141±14 bpm |
| (Ofner et al., 2014) | Ten healthy males  75.9±6.7 kg; 1.82±0.07m; 25.3±4.6 yrs | 3.90±0.49 l/min | Normobaric hypoxic  0 vs 3,500 m | cycle ergometer  40 watt for 3 minutes, followed by an increase of 20 watt per minute up to voluntary exhaustion | 190±8 bpm vs 185±7 bpm* |
| (Ogawa et al., 2010) | Eleven Trained subjects: 66±4 kg; 172±5 cm; 24±2yrs | 59.7±10.1 ml/kg/min | Hypobaric Hypoxia  485 vs. 2,500m | Treadmill  Between 160 or 220 m min^-1^ and then 20 m min^-1^ / 2 min | 186 ± 10 vs. 193 ± 8 bpm* |
| (Osawa et al., 2011) | Nine active male  66.0±5.7 kg; 174.1±6.7 cm; 23±2 yrs | 51.4±2.5 ml/kg/min | Normobaric hypoxia  0 vs 4,600 m | 10W then 20W every 1 min | 185±2 vs 178±2* |
| (Ponsot et al., 2010) | Fifteen male  31±2 yrs;  179±1 cm; 69.8±1.3 kg  Severe: 30 _2 yrs ; 68.9 _ 1,7 kg ; 180 _ 2 cm  Moderate: 33 _3 ; 70.6 _ 2.1 kg ; 178 _1 cm | endurance runners and triathletes 58.6±1.7 ml/kg/min  Devided  Severe loss of VO2max: 61.4 _ 1.7 ml/min  Moderate loss of VO2max: 56.2 _ 2.7 ml/min | Normobaric hypoxia  0 vs 3,000 m | Cycle ergometer  80 W then 40 W every 2 min | Severe Loss: 175 vs 173 bpm*  Moderate loss 174 vs 173 bpm |
| (Richalet et al., 1988) | Eight healthy subjects  32±8 yrs | 3.34±0.39 l/min | Hypobaric Hypoxia  1,035 vs 3,823m | Cycle ergometer | 189±11 vs 176±8 bpm* |
| (Rodway et al., 2016) | Eleven competitive male cyclists  67.2(63.4–71.0) kg, 165(160.5–169.5) cm, 66.5 (63.6–69.4) yrs | 39.1 (33.5–44.7) ml/min/kg | Hypobaric hypoxia  1,400 vs 2,750m | Cycle ergometer  Unloaded then then 25 W / min | 162.3(157.1–167.5) vs 163.9(153.6–174.2) bpm |
| (Roels et al., 2007) | Nineteen trained males  H: 73.2±0.8 and 71.3±0.9 kg; 180.1±0.5 cm  N: 181.6±0.7 cm; 24.4±0.3 and 24.2±0.4 yrs | H: 58.5±0.7 ml/kg/min  N:58.1±0.83 ml/kg/min | Normobaric hypoxia  0 vs 3,000 m | Cycle ergometer  60 W for 3 min then 30 W every min | Pre vs post training  H Pre: 189.7±1.1 vs 188.2±1.0 bpm  H Post: 189.4±1.0 vs 188.9±1.1 bpm  N Pre: 190.1±1.1 vs 187.6±1.4 bpm  N post: 188.3±1.5 vs 187.4±1.2 bpm  Hypoxic effect not tested |
| (Savoldelli et al. 2018; personal data) | Thirteen males  69.4±6.0 kg; 175.3±4.6 cm; 34.1±9.7 yrs | 3.8±0.5 L/min | Normobaric hypoxia  0 vs 3,500m | Cycle ergometer  75 W then 25 W / min | 177.5±12.6 vs 171.3±12.2 bpm* |
| (Schmidt et al., 1990) | Ten males  76.0 9.3 kg; 180 7 cm; 28.7 4.3 yrs | 47 ml/kg/min | Normobaric hypoxia  0 vs 3,800 m | Cycle ergometer  100 W then 17 W / min | 190 vs 189 bpm |
| (Shephard et al., 1988) | Eight males  70.2±8.7 kg; 181±6.7 cm; 28.6±3.8 yrs  Eight females  54.1±4.2 kg;163.3±4.0 cm;31.9±10.4 yrs | Males: 49.1±7.1 ml/kg/min  Females: 40.9±10.3 ml/kg/min | Normobaric hypoxia  0 vs 4,400 m | Cycle ergometer | Men: 180±13 vs 173±11 bpm*  Women:175±13 vs 168±14 bpm * |
| (Squires and Buskirk, 1982) | Twelve males  72.9±8.9 kg; 178.7±6.6 cm; 25.9±3.9 yrs | 3.25±0.11 ml/kg/min | Hypobaric hypoxia  362, 914, 1,219, 1,524, and 2,286 m | Treadmill  2.5% every 2 min | 362 m: 191.3±2.3  914 m: 191.3±2.4  1219 m: 190.5±2.3  1524 m: 190.4±1.8  2286 m: 187.9±2.7 |
| (Stenberg et al., 1966) | Six males  19-36 yrs | 3.46 L/min | Hypobaric Hypoxia  0 vs 4,000m | Cycle ergometer | 186 vs 184 bpm |
| (Subudhi et al., 2007) | Thirteen trained males (resident live between 1,585 and 1,890 m)  78±9 kg; 182±6 cm; 30±7 yrs | 60±75.6 ml/kg/min | Normobaric hypoxia  0 vs 4,500m | Cycle ergometer  25 W and then 25 W/min | 179±10 bpm vs H: 166±10 bpm* |
| (Subudhi et al., 2008) | Eleven males  77.6±12.3 kg; 176.5±7.5 cm; 21±3 yrs | 3.98±0.41 l/min | Normobaric hypoxia  0 vs 4,500m | Cycle ergometer  100, 130, and 160 W in 2-min then 15 W every min | SL: 187±9 bpm vs 181±7 bpm* |
| (Subudhi et al., 2011) | Thirteen trained males  76±11 kg; 180±8 cm; 32±8 yrs | 4.3±0.6 l//min | Hypobaric hypoxia  0 vs 4,875 m | cycle ergometer  30-min self-paced warm up followed by 25 W/min ramp) | N: 183±8 bpm vs H: 170±11 bpm* |
| (Torres-Peralta et al., 2015) | Eleven males  72.3±9.3kg; 174 ±8 cm; 21.5±2.0 yrs | 51±5 ml/kg/min | Normobaric hypoxia  0 vs 5,200m | cycle ergometer  N: 80W then 30W every 2min  H: 60W then 20W every 2 min | 185.4±6.0vs 175.1±9.0 bpm* |
| (Tymko et al. 2017) | Eleven males  71.1±2.5 kg; 176.3±1.5cm; 26.9±1.8 yrs | NA | Hypobaric hypoxia  344 m vs 3,800 m | Cycle ergometer  40 W for females, 60 W for males) then 20 W every 1min | 178 vs 170 bpm* |
| Valentini et al. 2012 | Twenty seven males  66.4±13.3 kg; 22.0±3.3 kg/m²; 39.1±12.7 yrs | 33.9±10.4 ml/kg/min | Hypobaric hypoxia  0 vs 4,559 m | Cycle ergometer  30 W/ min | 161.0±13.8 vs 142.1±21.4 bpm* |
| (Van Thienen and Hespel, 2016) | Eight trained and 7 untrained males  U: 67.0±1.9kg; 181.9±2.7; 24.0±1.9 yrs  T: 68.7±2.0; 180.0±2.6 cm; 25.4±1.8 yrs | U: 43.4±2.2 ml/kg/min  T: 70.0±1.7 ml/kg/min | Normobaric hypoxia  0 vs 5,300 m | Cycle ergometer  60 W + 30 W per 3 min | U: 195±4 vs 192±1 bpm  T: 187±4 vs 177±2 bpm* |
| (Ventura et al., 2003) | Twelve trained males  68.1±1.7 kg; 178.3±3.5cm; 25±2.2 yrs | 62.1±2.7 ml/kg/min | Normobaric hypoxia  560 m vs 3,200 m | Cycle ergometer  30 W every two min | H  Pre 188.6±3.1 vs 181.7±3.4 bpm  Post 184.9±3.1 vs 177.7±3.3 bpm  N  Pre 187.0±4.9 vs 181.2±4.2 bpm  Post 181.6±3.1 vs 176.0±3.3 bpm  Hypoxic effect not tested |
| (Vogiatzis et al., 2011) | Eleven trained males  71.5±7.6 kg, 1.8±0.3 m, 38±13 yrs | 62±7 ml/min/kg | Normobaric hypoxia  0 vs 4,350m | Cycle ergometer  30 W then 30 W / min | 183±17 vs 186±10 bpm |
| (Wehrlin and Hallen, 2006) | Eight trained males  72±4.2 kg; 181±3.5 cm; 24.4±3.5 yrs | 66±4.3 ml/kg/min | Hypobaric hypoxia  300, 800, 1,300, 1,800, 2,300, 2,800m | Treadmill  14.3±1.5 km/h then 1 km/h per min | 300 m: 189 bpm  800 m: 187 bpm  1300 m: 188 bpm  1800 m: 185 bpm  2300 m: 185 bpm  2800 m: 184 bpm  Overall effect of altitude |
| (West et al., 1983) | Fifteen males  74.9+8.3 kg; 179.3+6.2 cm; 36.5+8.0 | 4.63 l/min | Hypobaric hypoxia  0 vs 6,300 m | Cycle ergometer  300 kg/m/min then increase / 3 min | 178 vs 146 bpm |
| (Woorons et al., 2005) | Seven trained females  55.9±6.4 kg; 165.7±7.2 cm; 29.8±5.0 yrs  Seven untrained females  61.4±9.9 kg; 166.0±6.7 cm; 25.9±4.4 yrs | U: 34.8±5.6 ml/kg/min  T: 56.3±4.7 ml/kg/min | Normobaric hypoxia  1000, 2500 and 4,500m | Cycle ergometer  45 W then 25 W / min | U  SL: 188±8 bpm  1000 m: 185±7 bpm*  2500 m: 186±5 bpm  4500 m: 181±6bpm*T  SL: 179±8 bpm  1000 m: 176±11 bpm*  2500 m: 175±10 bpm*  4500 m: 167±11 bpm* |
| (Young et al., 1982) | Eight males  75.5± 2.5 kg; 175.6± 2.2cm; 23.3± 1.3 yrs | 3.39±0.11 l/min | Hypobaric hypoxia  0 vs 4,300 m | Cycle ergometer  Discontinuous cycling test | 182±3 vs 166±6 bpm* |
| **Patients** | | | | | |
| (Erdmann et al., 1998) | Twenty-three patients with coronary artery disease  27±3 kg/m²; 51±9 yrs  Twenty-three healthy subjects  26±4 kg/m²; 53±6 yrs | NA | Hypobaric Hypoxia  1,000 m and 2,500m | Cycle ergometer  60 W then 30 W every 2 min | Controls: 157±16 vs 156±16 bpm  Patients: 156± 18 vs 155±19 bpm |
| (Garcia et al., 1999) | Eleven Fontan patients  14.5+/-5.2 yrs | 23.5±5.3 ml/kg/min | Hypobaric hypoxia  0 vs 3,048 m | Cycle ergometer  Modified James protocol | 161.8 13.2 vs 161.9 17.9 |
| (Schmid et al., 2006) | Twenty two coronary patients  26±4 kg/m²; 57± 7 yrs | 28.3±4.4 ml/kg/min | Hypobaric hypoxia  540 and 3,454 m | Cycle ergometer  15 W every 2 min | 163±11 vs 159±11 bpm* |
| (Schmid et al., 2015) | Twenty nine Heart failure patients  60.0 ± 8.9 yrs | 18.5±3.6 ml/kg/min | Hypobaric hypoxia  540 vs 3,454 m | Cycle ergometer  15 W every 2 min | 124.3±24.0 vs 132.7±24.3 bpm  **Significantly HIGHER in Hypoxia** |
| (Staempfli et al., 2016) | Seventeen Fontan patients  22.5±2.1 kg/m²; 27±7 yrs  Fifteen healthy controls  22.7±3.5 kg/m²; 29±8 yrs | Fontan patients: 22.8±5.1 ml/kg/min  healthy controls: 35.0±7.4 ml/kg/min | Hypobaric hypoxia  540 m vs 3,454 m | Cycle ergometer  Ramp protocol | Fontan patients  172 (131, 178) vs 164 (132,172) bpm*  Healthy controls  182±13 vs 177±13 bpm* |

U= untrained subjects; T=Trained subjects; H = Hypoxia; N = normoxia; EIH = exercise induced hypoxemia, bpm= beat per minute. NA= not available *= Significantly different from SL. Low altitude was set at 0 m unless specified.

**Table S2:** Exercise intervention programs in normoxia or hypoxia based on percentage of maximal heart rate or heart rate reserve

| Study | Participants | Training status | Training program | Main findings |
| --- | --- | --- | --- | --- |
| Healthy subjects | | | | |
| (Bailey et al., 2000) | Thirty four males  N: 74.5±6.3 kg; 1.76±0.05 cm; 22±1 yrs  H: 75.2±8.3 kg; 1.79±0.07 cm; 22±3 yrs | N: 61.2±10.9 ml/min/kg  H: 54.1±9.5 ml/min/kg | Normobaric hypoxia  0 vs 2100m  20-30 min cycling 3 times / week for 4 weeks.  70, 75, 80, and 85% of HR_max_ determined in the corresponding environment. | Significant improvements in selected risk factors and exercise capacity for both N and H. Additive cardioprotective effect with H. |
| (Dufour et al., 2006) | Eighteen male runners divided  N: 71.3±2.2 kg; 180±2 cm; 30.3±6.1 yrs  H: 70.6±2.2 kg; 180±1 cm; 30.3±6.3 yrs | N: 61.5±1.1 ml/min/kg H:64.2±1.2 ml/min/kg | Normobaric hypoxia  300 vs 3000m  6 weeks of running training with 5 sessions per week including 2 specific sessions at the relative VT2. No significant difference in absolute training HR. | Test in hypoxia: improvement of maximal aerobic velocity and VO_2max_ only in H.  Test in normoxia: improvement of maximal aerobic velocity in both groups. Improvement of VO_2max_ for H group only. |
| (Hamlin et al., 2010) | Sixteen athletes  N: 76.7±12.3kg; 1.78±0.1 m; 38.7±8.6 yrs  H: 76.6±11.1kg; 1.72±0.1 cm; 29.6±12.3 yrs | NA | Normobaric hypoxia  0 vs 3,200-4,000-4,400m  10 consecutive days 90 min at 60–70% of HRR in normoxia  Regression equation that describes the decrease in chronotropic drive during hypoxia, y (% of sea-level HR_max_)=116–0.0057x (=altitude in meters, or *S*aO_2_) was used to predict HR_max_ in H (Richalet, 1992) | Significantly higher increase in 30 sec mean power but not 30 sec peak power, 20km mean power and 20 km energy cost. |
| (Haufe et al., 2008) | Twenty healthy males  H: 82.5±12.0 kg; 1.8±0.1cm; 29±5.9 yrs  N:78±7.5 kg; 1.8±0.1cm; 28.1±5.2 yrs | N: 44±6.2 ml/min/kg  H: 47±3.4 ml/min/kg | Normobaric hypoxia  0 vs 2,740m  60 min 3 times/ week for 4 week  HR at 3 mmol/L blood lactate in corresponding environment | Similar or even better response in terms of cardiovascular and metabolic risk factors in H than H |
| (Hendriksen and Meeuwsen, 2003) | Fourteen healthy triathletes  H: 72 (61-85) kg; 182 (175-191) cm; 30.3 (21-39) yrs  N:80 (77-87) kg; 190 (183-198) cm; and 29.0 (21-39) yrs | N: 66.3±5.2 ml/min/kg  H: 64.4±5.9 ml/min/kg | Hypobaric hypoxia  0 vs 2,000 m then 3,000 m  105 min cycling 10 consecutive days.  60-70% of HRR based on HR_max_ obtained at sea level and HR rest measured the morning of the training session. | Significant improvement of both aerobic and anaerobic variables including maximal power |
| (Mao et al., 2011) | Twenty-four healthy sedentary males  N: 68.1±2.2kg; 172±1 cm; 22±1 yrs  H: 65.9±2.5kg; 173±1 cm; 22±1 yrs | N: 50.9±2.5 ml/min/kg  H: 50.3±2.3 ml/min/kg | Normobaric hypoxia  0 vs 2,733 m  30 min per session, 5 times / week for 5 weeks  65%HRR obtained in corresponding environment | H, not N, promoted eryptotic response to oxidative stress. H attenuates the extents of erythrocyte deformability and dehydration modulated by Gardos channel. |
| (Masuda et al., 2001) | Fourteen males  N: 58.0±3.8 kg; 168.0±6.0 cm; 20.3±1.1 yrs  H: 59.6±7.3 kg; 169.9±15.5 cm; 119.9±1.1 yrs | N: 44.7±9.7 ml/min/kg H:45.3±7.8 ml/min/kg | Hypobaric hypoxia  0 vs 2,500m  1h per training session, 28 sessions over 8 weeks.  Target HR corresponding to HR at 60 then 70% VO_2max_ obtained in corresponding environment | No significant difference in myoglobin content |
| (Meeuwsen et al., 2001) | Fourteen healthy triathletes divided  N: 80 (71-87) kg; 188 (180-198) cm; 28.3 (21-39) yrs  H: 72 (61-85) kg; 182 (175-191) cm; 29.6 (21-39) yrs | N: 3.6±5.1 ml/min/kg H:67.1±3.8 ml/min/kg | Hypobaric hypoxia  0 vs 2,500 m  2h cycling 10 consecutive days  60-70% of HRR based on max HR obtained at sea level and HR rest measured the morning of the training session. | Significant improvement in VO_2max_ and maximal power only in H.  Significant increase in mean and peak power during Wingate test only in H |
| (Pesta et al., 2011) | Twenty-five healthy sedentary males  N: 91.10±14.6 kg; 183.1±7.2 cm; 27.8±7.4 yrs  H: 89.6±21.4 kg; 181.8±5.9 cm; 28.8±7.0 yrs | N: 38.0±7.7 ml/min/kg  H: 41.5±8.6 ml/min/kg | Normobaric hypoxia  0 vs 4,000 m  3 times / week for 10 weeks  two continuous (35-55 min) sessions at 75% HR_max_ and one session of 2-3 sets (3’ with 2’rec) of interval training at 95% of HR_max_ obtained in Normoxia | No significant effects between the two groups |
| (Shi et al., 2013) | Eight healthy men 71.4±14.5 kg ; 174.9 ± 5.3 cm; 25.8±7.8 yrs | NA | Normobaric hypoxia  0 vs 2,500m  50 min 3 times / week for 4 weeks  60% of HR_max_ in normoxia | Significant decreases in preperitoneal fat and Brachial ankle pulse wave velocity, and significant increase in VO_2max_ in H compared to N. |
| (Shi et al., 2014) | Fourteen healthy men (27.4±2.6 yrs)  14 N: 70.3±3.1kg; 174.4±1.5 cm  14H: 70.2±3.1kg; 174.4±1.5 cm | N: 54.1±2.5 ml/min/kg  H: 51.7±2.4 ml/min/kg | Normobaric hypoxia  0 vs 2,500m  50 min 3 times / week for 4 weeks  60% of HR_max_ in normoxia | Significantly greater changes in waist circumference, preperitoneal fat thickness, brachial-ankle pulse wave velocity, and high-sensitivity in H than in N |
| (Wang et al., 2014) | Forty healthy sedentary males  N: 67.0±1.7 kg; 173.5±1.4 cm; 21.7±0.3 yrs  H: 66.8±2.1 kg; 172.7±1.2 cm; 21.8±0.8 yrs | N: 42.8±0.9 ml/min/kg  H: 43.5±1.1 ml/min/kg | Normobaric hypoxia  0 vs 2,200 m  4 times / week for 3 weeks  55-65%HRR obtained in normoxia | Significant greater improves cardiac and muscular hemodynamic adaptations in H than N |
| Patients | | | | |
| (Gatterer et al., 2015) | Thirty-two healthy sedentary males  N: 103.2±15.1kg; 36.3±4.2 kg/m²  H: 105.5±20kg; 37.9±8.1 kg/m² | N: 19.1±5.8 ml/min/kg  H: 19.3±6.2 ml/min/kg | Normobaric hypoxia  130 and 490m vs 3,500 m  90 min per session, 2 times / week for 8 months  65-70%HRR obtained in normoxia  + 90 min rest (130 or 490 vs 4,500m), 2 times / week | Long-term, moderate intensity exercise and rest in H does not lead to higher reductions in body weight than N alone |
| (Greie et al., 2006) | Seventy one male non-smoking subjects with the metabolic syndrome divided in two groups  56 (36-66 years). | NA | Hypobaric hypoxia  500 vs 1,700 m  30 min per session, 5 times / week for 5 weeks  55-65%HRR obtained in normoxia | No significant effects between the two groups |
| (Gutwenger et al., 2015) | Fourteen patients with metabolic Syndrome  N: 88.6±20.2 kg, 50.1± 7.8 years  H: 91.8±22.5 kg, 63.3±5.2 years | N: 1754±383.8 ml/min  H: 2335±1058.4 ml/min | Hypobaric hypoxia  300 vs 1,900 m  Hiking three hours a day, four times a week (total training time, 24 hours) at an intensity of 55–65% HR_max_ obtained in Normoxia | 2-week hiking vacation at moderate altitude may be more beneficial for adipokines and parameters of lipid metabolism than training at low altitude. |
| (Kong et al., 2014) | Eighteen obese young adults  N: 103.4±24.7 kg; 33.8 ±5.6 kg/m²; 22.3±1.7 yrs  H: 99.0±19.5 kg; 34.7±5.3 kg/m²; 19.8 ±2.2 yrs | NA | Normobaric hypoxia  0 vs 2,000 then 3,000m  3 sessions / week for 4 weeks  60–70 % of predicted HR_max_ (220−age) | Weight reduction significantly larger in H |
| (Netzer et al., 2008) | Twenty overweight subjects  body mass index >27 kg/m²; 47.6 yrs | NA | Normobaric hypoxia  450 vs 2,500 m  3 sessions / week for 8 weeks  90 min at 60% of HR_max_ in normoxia | Weight reduction significantly larger in H |
| (Nishiwaki et al., 2011) | Sixteen postmenopausal women  55.7±1.5 kg; 151.9±1.6 cm; 56±1 yrs | N: 25.0±1.9 ml/min/kg  H: 27.2±2.6 ml/min/kg | Hypobaric hypoxia  0 vs 2,000m  30 min per training session, 4 days per week, for 8 weeks.  Target HR corresponding to HR at 50% VO_2max_ obtained in Normoxia. | No significant change in resting HR, blood pressure and VO_2max_ in normoxia. Mean brachial–ankle pulse wave velocity significantly reduced in H, but not in N. Flow-mediated vasodilation significantly increased in H only |
| (Park and Lim, 2017) | Thirty-six middle-aged obese women in 3 groups  65.6±7.4 kg; 57.7± 3.9 cm; 47.2 ± 6.3 years  62.8 ± 8.9 kg; 157.6 ± 6 cm; 42.0 ± 4.4 years  64.6 ± 9.7 kg; 158.3 ± 5.3 cm; 46.6 ± 5.7 years | NA | Normobaric hypoxia  0 vs 2,000m vs 3,000m  1 hour, 5 times / week for 6 weeks  75% predicted HR_max_ (205 - 0.75 × age). | Significantly greater changes in body composition, blood pressure, arterial stiffness and blood lipid with H |
| (Pramsohler et al., 2017) | Nineteen elderly patients with various diseases N: 25.8±6.2; kg/m²; 82.0±7.8 yrs  H: 25.2±5.3 kg/m²; 80.2±7.2 yrs | N: 938.13±218.01 ml/min  H: 929.68±325.6 ml/min | Normobaric hypoxia  0 vs 2,000m  7 sessions during 3 weeks  HR achieved at 80% VO_2max_ in incremental test in normoxic conditions, but significantly higher HR in H | No significant difference |
| (Schreuder et al., 2014) | Nineteen type 2 diabetes patients  H: 106±18 kg; 181±10 cm; 57±6 yrs  N: 115±23 kg; 179±6 cm; 52±8 yrs | N: 24.2±7.1 ml/min/kg  H: 27.3±4.7 ml/min/kg | Normobaric hypoxia  0 vs 2,000m  45 min cycling per training session, 3 times a week for 8 weeks. 70%–75% of HRR obtained in Normoxia. | No significant difference physical fitness, vascular function, or glucose homeostasis |
| (Wiesner et al., 2010) | Forty-five non-diabetic or insulin-resistant, overweight or obese:  30.2±3.6 kg/m^2^; 42±7.1 yrs | N: 32±1.2 ml/min/kg  H: 36±1.5 ml/min/kg | Normobaric hypoxia  0 vs 3,500m  60 min 3 days / week for 4 weeks  HR at 65% of VO_2max_ in normoxia | Similar or even better response in terms of physical fitness, metabolic risk markers, and body composition |

H = Hypoxia; N = normoxia. NA= not available
